# Phenomic screen identifies a role for the yeast lysine acetyltransferase NuA4 in the control of Bcy1 subcellular localization, glycogen biosynthesis, and mitochondrial morphology

**DOI:** 10.1371/journal.pgen.1009220

**Published:** 2020-11-30

**Authors:** Elizabeth A. Walden, Roger Y. Fong, Trang T. Pham, Hana Knill, Sarah Jane Laframboise, Sylvain Huard, Mary-Ellen Harper, Kristin Baetz

**Affiliations:** 1 Department of Biochemistry, Microbiology and Immunology, Faculty of Medicine, University of Ottawa, Ottawa, Canada; 2 Ottawa Institute of Systems Biology, Ottawa, Canada; The University of North Carolina at Chapel Hill, UNITED STATES

## Abstract

Cellular metabolism is tightly regulated by many signaling pathways and processes, including lysine acetylation of proteins. While lysine acetylation of metabolic enzymes can directly influence enzyme activity, there is growing evidence that lysine acetylation can also impact protein localization. As the *Saccharomyces cerevisiae* lysine acetyltransferase complex NuA4 has been implicated in a variety of metabolic processes, we have explored whether NuA4 controls the localization and/or protein levels of metabolic proteins. We performed a high-throughput microscopy screen of over 360 GFP-tagged metabolic proteins and identified 23 proteins whose localization and/or abundance changed upon deletion of the NuA4 scaffolding subunit, *EAF1*. Within this, three proteins were required for glycogen synthesis and 14 proteins were associated with the mitochondria. We determined that in *eaf1*Δ cells the transcription of glycogen biosynthesis genes is upregulated resulting in increased proteins and glycogen production. Further, in the absence of *EAF1*, mitochondria are highly fused, increasing in volume approximately 3-fold, and are chaotically distributed but remain functional. Both the increased glycogen synthesis and mitochondrial elongation in *eaf1*Δ cells are dependent on Bcy1, the yeast regulatory subunit of PKA. Surprisingly, in the absence of *EAF1*, Bcy1 localization changes from being nuclear to cytoplasmic and PKA activity is altered. We found that NuA4-dependent localization of Bcy1 is dependent on a lysine residue at position 313 of Bcy1. However, the glycogen accumulation and mitochondrial elongation phenotypes of *eaf1Δ*, while dependent on Bcy1, were not fully dependent on Bcy1-K313 acetylation state and subcellular localization of Bcy1. As NuA4 is highly conserved with the human Tip60 complex, our work may inform human disease biology, revealing new avenues to investigate the role of Tip60 in metabolic diseases.

## Author summary

Metabolism, how cells process nutrients and energy substrates, is dramatically altered in many diseases. For example, during tumorigenesis, changes in cellular metabolism allow for the rapid cellular growth and division. One way to control metabolism is by moving the proteins involved in metabolism to different parts of the cell. Similar to an assembly line, when all the “parts” (metabolic proteins) are brought together, efficiency can be increased. In contrast, if one or more parts are shipped to the wrong place (mislocalized), production is decreased, and an alternative production process may even be necessary to meet demands. Here we ask whether Tip60, a protein complex implicated in many diseases, affects the location of metabolic proteins in the cell. We use budding yeast as a model system to assess the effect of disrupting yeast Tip60 on the location of over 360 metabolic proteins. We found that Tip60 controls the location of many metabolic proteins and in the absence of Tip60 there is an increase in the mitochondria and in cellular carbohydrate storage (the metabolic ‘hubs’ of the cell). This work suggests that yeast Tip60 functions as a “brake” on cellular metabolism and provides novel potential roles of Tip60 in metabolic diseases.

## Introduction

Lysine acetylation is one of the most common post-translational modifications (PTM) in the cell. Lysine acetylation has been historically characterized as a PTM occurring on histones, however, in the last 10 years, thousands of non-histone acetylation sites have been identified through global screens for protein acetylation, establishing lysine acetylation as a conserved and dynamic PTM [1–6]. It is therefore not surprising that lysine acetylation plays diverse roles in the regulation of cellular processes and that it is deregulated in many human diseases [7–10].

Lysine acetylation is catalytically controlled by two opposing classes of enzymes: Lysine acetyltransferases (KATs) and lysine deacetylases (KDACs). Our work focuses on the *Saccharomyces cerevisiae* KAT complex NuA4, the yeast homolog of the human Tip60 complex, which has been associated with many diseases including cancer, developmental issues, and neurodegenerative disease [9,11–15]. Given the exceptional conservation of KAT function across eukaryotes, the study of the homologous NuA4 complex may provide insight into further understanding the biological roles of Tip60 and the cellular consequences of its deregulation in disease [11].

NuA4 contains 13 subunits, six of which are essential genes for viability, including the gene for the catalytic subunit *ESA1* [16–19]. Though non-essential, the *EAF1* subunit is a critical scaffolding protein required to maintain the complete NuA4 complex [16,19]. Yeast cells deficient for Eaf1 are viable but lose much of the targeting of the catalytic Esa1 subunit, making the *eaf1Δ* mutant an excellent genetic model system to identify the biological roles and protein targets of NuA4 [16,18,20,21]. Alternatively, NuA4 function can be probed using the temperature sensitive mutation, *esa1-ts* (*esa1-L245P*), which reduces the acetylation activity of Esa1 at increased temperatures [2,17,22].

Given its essential role, it is not surprising that NuA4 has been implicated in a myriad of biological processes, including metabolism. Indeed, lysine acetylation, which neutralizes the positive lysine side chain charge, has been suggested to be an ideal “switch” to turn off and on enzymes. For example, NuA4 dependent acetylation of phosphoenolpyruvate carboxykinase (Pck1) is important for activating its function in the rate controlling step of gluconeogenesis [23–25]. Acetylation has also been implicated in controlling protein-protein interactions. NuA4 yeast mutants have replicative lifespan defects caused by impairments in the NuA4-dependent acetylation of Sip2 [26]. Sip2 acetylation increases its interaction with Snf1 decreasing Snf1 activity [26]. Sip2 is one of the three inhibitory β subunits of the Snf1 complex (AMPK in humans) [27,28]. The Snf1 kinase responds to changes in cellular AMP levels and plays an important role in the adaptation of yeast cells to glucose limiting conditions as its kinase activity leads to the derepression of many glucose repressed genes [26–30]. Similarly, work by Filteau and colleagues suggests that the protein-protein interactions which control the activity of the Protein Kinase A (PKA), also referred to as cAMP-dependent protein kinase, are regulated through NuA4-dependent acetylation of the PKA regulatory subunit Bcy1 [31]. PKA is a central metabolic kinase and has been extensively linked to many cellular processes including stress response and mitochondrial processes [32–35]. In yeast, the PKA complex is a heterotetrameric complex containing two Bcy1 regulatory subunits bound to two catalytic subunits, of the three partially redundant Tpk1, Tpk2, and Tpk3 [36–39]. The study by Filteau and colleagues suggests that the unacetylated form of Bcy1 can interact with and inhibit the catalytic PKA subunits Tpk1/2/3, but when Bcy1 is acetylated the Bcy1-Tpk interaction is disrupted leading to PKA activation [31]. In humans, PKA activity can be regulated through being anchored to different parts of the cell including the mitochondria by A-Kinase Anchoring Proteins (AKAPs) [40]. While AKAPs have not been identified in yeast, the localization of the Tpks and Bcy1 are regulated in response to environmental factors which is likely also associated with changes in activity or substrates [34,41,42]. One key downstream effect of Tpk activity is the regulation of the localization of the functionally redundant Msn2 and Msn4 (Msn2/4) stress response transcription factors which are phosphorylated and sequestered in the cytoplasm [43,44]. Upon stress or inhibition of PKA activity, Msn2/Msn4 phosphorylation decreases allowing for translocation into the nucleus where they bind to stress response elements (STREs) and activate transcription of genes related to β oxidation of fatty acids, glycolysis, and stress response [45–47]. Interestingly, Msn2/4-dependent transcription is de-repressed in NuA4 mutants [20]. While this de-repression may be associated with PKA activity, it is also possible that there is direct regulation as NuA4 can co-IP with Msn2 and Msn4 [20,44]. Finally, PKA activity and transcriptional activation of Msn2/4 targets have also been linked to another target of NuA4, the oxysterol binding protein Kes1 [48]. Kes1 functions as a lipid exchange protein in the cell and it’s acetylation has been demonstrated to regulate cell cycle arrest in response to nutrient stress [48]. Yeast with impaired NuA4 activity showed increased Kes1 activity with an associated increase in Msn2/4 transcriptional activity in addition to the metabolic characteristics of quiescence [48]. Overall, many preliminary links of NuA4 to metabolism have begun to be uncovered.

In addition to regulating enzyme activities and protein-protein interactions directly, lysine acetylation has also been associated with contributing to protein subcellular localization. For example, in mammalian cells, Tip60 acetylation of lipin drives its localization to the endoplasmic reticulum [49]. Indeed, the role of acetylation in regulating protein subcellular localization maybe far reaching. Chong and colleagues used synthetic genetic array (SGA) technology coupled with high content screening to assess the impact of the deletion of the KDAC *RPD3* on over 4000 GFP fusions [50]. *RPD3* deletion led to a large number of proteins that increased in abundance but more intriguingly, the subcellular localization of more than 30 proteins were altered upon deletion of *RPD3* [50].

Given that NuA4 is implicated in the regulation of a variety of metabolic proteins [25,51], and the global impact of Rpd3 on protein abundance and localization [50], here we ask if NuA4 impacts the subcellular localization or abundance of metabolic proteins. We identified 23 proteins that displayed altered localization or abundance upon deletion of *EAF1* and of these, 14 were associated with mitochondria and three were associated with glycogen synthesis. We determined that the mitochondrial elongation and increased glycogen detected in *eaf1Δ* cells are partially due to the yeast PKA regulatory subunit, Bcy1, and that NuA4 is regulating the subcellular localization of Bcy1.

## Results

### *EAF1* dependent remodeling of the metabolic proteome

To identify metabolic protein localization and abundance changes between wild type (WT) and the NuA4 mutant *eaf1Δ* we performed a focused phenomic screen. Using synthetic genetic array technology [52,53], both WT and *eaf1Δ* strains were crossed to 407 C-terminal GFP-fusion proteins from the GFP collection [54] with known or implicated roles in metabolism. Due to attrition of strains during mating, this resulted in a pair matched single (X-GFP) and double (X-GFP *eaf1Δ*) mutant set consisting of 368 genes (Screen outlined in S1 Fig).

**Fig 1:**
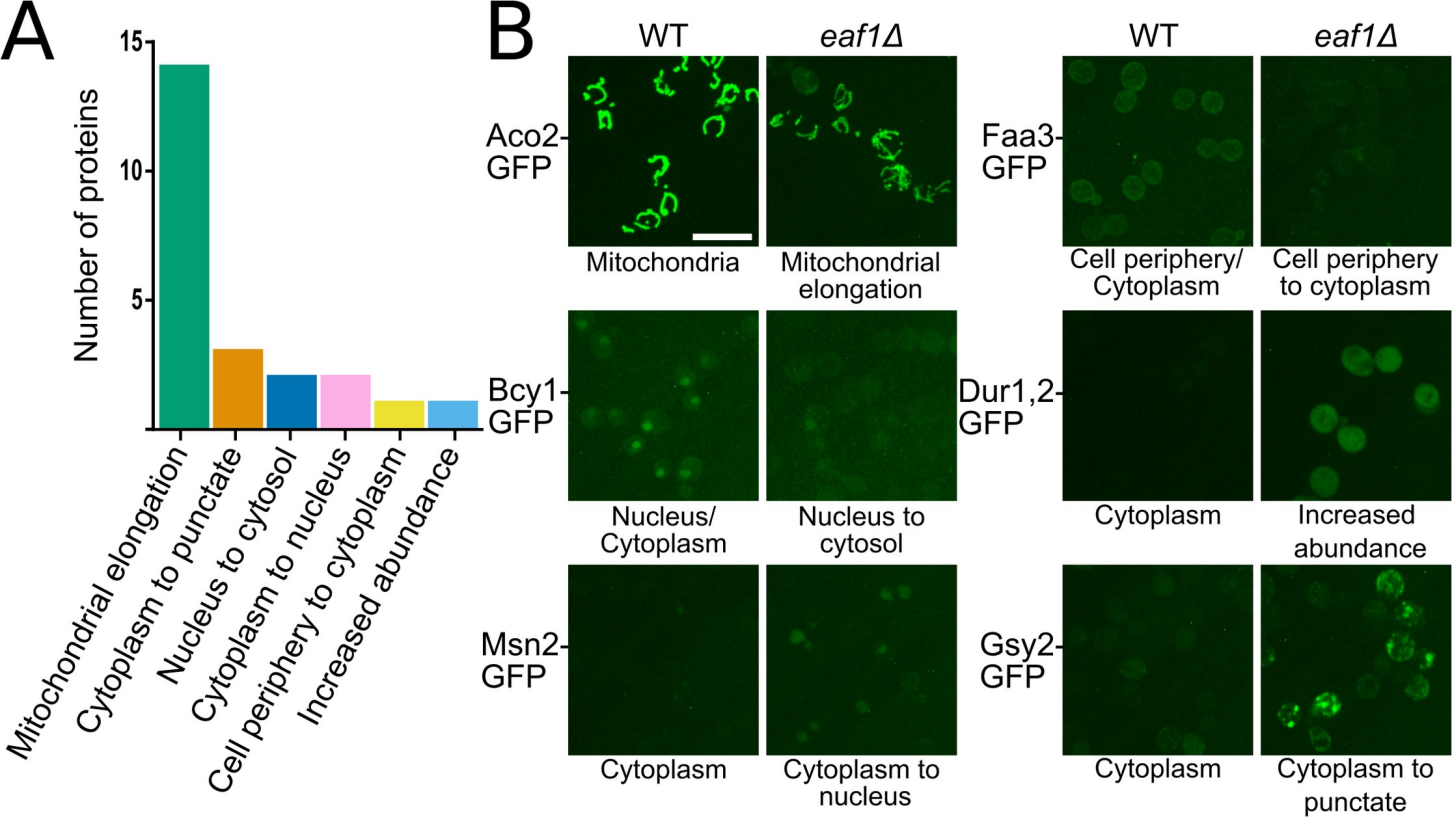
Summary of impact of *EAF1* deletion on metabolic protein localization and signal. (A) A phenomic screen for changes in protein localization and abundance between WT and *eaf1Δ* yeast was performed using SGA technology and high throughput microscopy. 23 confirmed changes in GFP signal localization or abundance were identified and categorized (S2 Table). (B) Six representative images of the change in GFP-fusion proteins identified in the phenomic screen (images of all confirmed changes available in S3 Fig). WT localization of proteins as previously reported at https://thecellvision.org/cyclops/ are listed under the WT image and the change category is listed under the *eaf1Δ* image. Some of these proteins also had changes in abundance in the *eaf1Δ* relative to WT. Fold changes outside our cut-off were: Aco2 = 0.51, Dur1, 2 = 3.77, and Gsy2 = 1.65. Scale bar = 10 μm.

The localization and protein abundance of the 368 GFP-tagged metabolic proteins was assessed in the WT and *eaf1Δ* background by high-throughput microscopy. In total, manual classification identified 70 proteins whose protein level and/or localization is impacted by deletion of *EAF1*. Additionally, the GFP intensity between WT and *eaf1Δ* cells for each strain was compared using ImageJ to assess changes in protein abundance, many of which corresponded with the manual classifications (S1 Table and S2 Fig). Of the approximately 70 potential changes identified (manually and by Image J), we selected 23 GFP fusions that had dramatic observable changes in localization upon deletion of *EAF1* to reassess. The identity of the selected GFP fusions were confirmed by PCR in both WT and *eaf1Δ* backgrounds and secondary microscopy was performed (Figs 1 and S3 and S1 Table). The 23 confirmed changes were categorized into six categories: mitochondrial elongation, cytoplasm to punctate, nucleus to cytoplasm, cytoplasm to nucleus, cell periphery to cytoplasm, and increased abundance (Figs 1A and S3 and S2 Table). Two categories of localization changes particularly stood out, the cytoplasm to punctate phenotype and the mitochondrial elongation phenotype. The cytoplasm to increased punctate phenotype (represented by Gsy2-GFP in Fig 1B) was of interest because all three proteins within this category are part of the glycogen regulation pathway. The second striking feature of this screen were the 14 GFP-tagged mitochondrial proteins, which displayed elongated mitochondrial phenotype (represented by Aco2-GFP in Fig 1B). Our screen shows that deletion of *EAF1* results in remodeling of a subset of the yeast metabolic proteome.

### NuA4 regulation of glycogen biosynthesis is dependent on PKA and Msn2/Msn4

**Fig 2:**
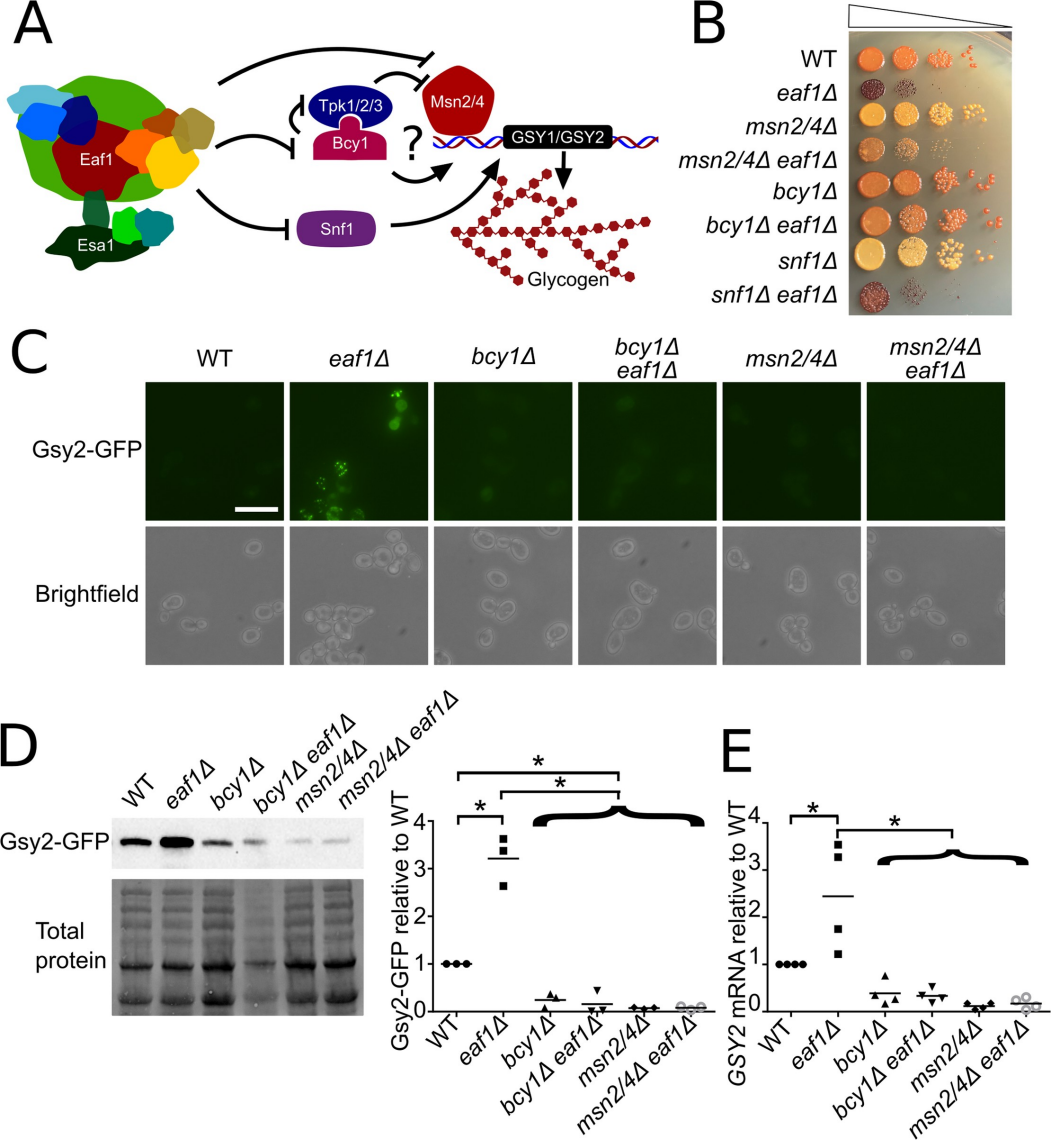
NuA4 regulation of glycogen biosynthesis proteins is mediated by PKA-Msn2/4 pathway. (A) Schematic model of NuA4 regulation of glycogen biosynthesis genes. (B) Increased glycogen levels in *eaf1Δ* cells is suppressed by *bcy1Δ* and *msn2Δmsn4Δ*. The indicated strains were serially diluted on to YPD plates, grown at 30°C for 24 hours prior to exposure to iodine crystal vapours. Darker color corresponds to more glycogen storage. (C) Gsy2-GFP expression and localization were assessed in the indicated strain backgrounds by microscopy. Images are representative of three independent biological replicates. Scale bar = 10 μm. (D) Gsy2-GFP protein levels were assessed by quantitative western blot analysis using whole cell extracts from the indicated strains expressing Gsy2-GFP. Panel to the left is representative anti-GFP western and total protein blot; panel to the right is the quantification of western blots for three independent biological replicates where the Gsy2-GFP band intensity was normalized to total protein. (E) *GSY2* mRNA was measured using RT-qPCR relative to the *TDH3* gene for indicated yeast strains using four biological replicates. For D and E, ANOVA analysis was performed with a Tukey’s multiple comparison test comparing pairs of means. * = p < 0.05, relevant significance bars shown. Horizontal bar in data represents the mean.

Our screen identified three GFP-tagged glycogen biosynthesis proteins (Gdb1, Gsy1, and Gsy2) that in *eaf1Δ* cells increased in GFP signal and were concentrated into punctate structures (Figs 1B, S3 and S4A). These proteins are responsible for glycogen synthesis (Gsy1 and Gsy2) and glycogen debranching (Gdb1) [55]. Glycogen is a large polysaccharide molecule made of repeating and branching glucose molecules [55] (Fig 2A). It is an important energy storage molecule for mammals and yeast [55–57]. Iodine staining to assess glycogen content confirmed that *eaf1Δ* cells have a higher level of glycogen (darker staining) than WT cells [58] (Fig 2B). Additionally, at the semi-restrictive temperature of 30°C the temperature sensitive *ESA1* mutant *esa1*^*L254P*^ *(esa1-ts*) also demonstrated an increase in iodine staining (S4 Fig).

We next sought to determine mechanistically how deletion of *EAF1* causes an increase in glycogen biosynthesis proteins. Given the similar induction in protein levels observed by microscopy and western blot (Figs 2C, 2D and S4) and that *GDB1*, *GSY1*, and *GSY2* genes are all associated with stress response element (STRE) activated transcription [46,59–61], we performed further characterization using only Gsy2, the predominant glycogen synthase in yeast [62]. As both the PKA-Msn2/Msn4 axis and Snf1 have been implicated in the transcriptional regulation of glycogen biosynthesis genes [59,60,63,64] (Fig 2A) we asked if the increased glycogen level seen in *eaf1Δ* cells is dependent on *MSN2/MSN4*, *BCY1*, or *SNF1* (Fig 2B). We found that *eaf1Δ* cells have a much darker iodine staining than WT cells, suggesting an accumulation of glycogen (Fig 2B). While *snf1*Δ cells displayed less iodine staining than WT cells, suggesting less glycogen accumulation, *snf1ΔeafΔ* cells displayed iodine staining similar to *eaf1Δ* cells. In contrast, deletion of both *MSN2* and *MSN4* (*msn2/4Δ)* or deletion of *BCY1* (*bcy1Δ)* suppressed glycogen accumulation in *eaf1Δ* cells when analyzed by iodine staining (Fig 2B). This suggests that NuA4-dependent regulation of the PKA-Msn2/4 pathway contributes to the control of glycogenesis, while NuA4-dependent regulation of AMPK/Snf1 does not. Further, we were surprised to see that deletion of *BCY1* suppressed the slow growth defects of *eaf1Δ* cells, where *msn2Δmsn4Δ* did not (Fig 2B). The *bcy1Δ* suppression of *eaf1Δ* slow growth was confirmed by OD-based growth curve analysis and calculation of doubling time for each strain (S5 Fig). This suggests that derepression of Msn2/Msn4-dependent transcription is not contributing to slow growth of *eaf1Δ*, rather other targets of PKA are likely contributing to its slow growth. The dependence of the glycogen content on Msn2/4 signalling was of additional interest to us as our screen also identified Msn2 to move from the cytoplasm to the nucleus upon deletion of *EAF1* (Fig 1B). Parallel analysis of the Gsy2-GFP signal by microscopy and quantitative western blot confirmed that Gsy2-GFP protein expression is induced upon deletion of *EAF1* and demonstrated that this is dependent on Msn2/Msn4 and Bcy1 (Fig 2C and 2D). qRT-PCR determined that the increase in Gsy2 protein was at least partially due to a significant increase in *GSY2* mRNA expression in *eaf1Δ* cells relative to WT (Fig 2E). Finally, the increase in protein abundance of Gsy2-GFP and mRNA levels of *GSY2* seen in *eaf1*Δ cells were suppressed upon deletion with *msn2Δmsn4*Δ or *bcy1Δ (*Fig 2C, 2D and 2E). Together this work is consistent with the conclusion that the increase in glycogen biosynthetic proteins identified in our screen are at least partially due to NuA4 regulation of PKA and Msn2/Msn4 activity.

### Mitochondrial volume increases in the absence of NuA4

**Fig 3:**
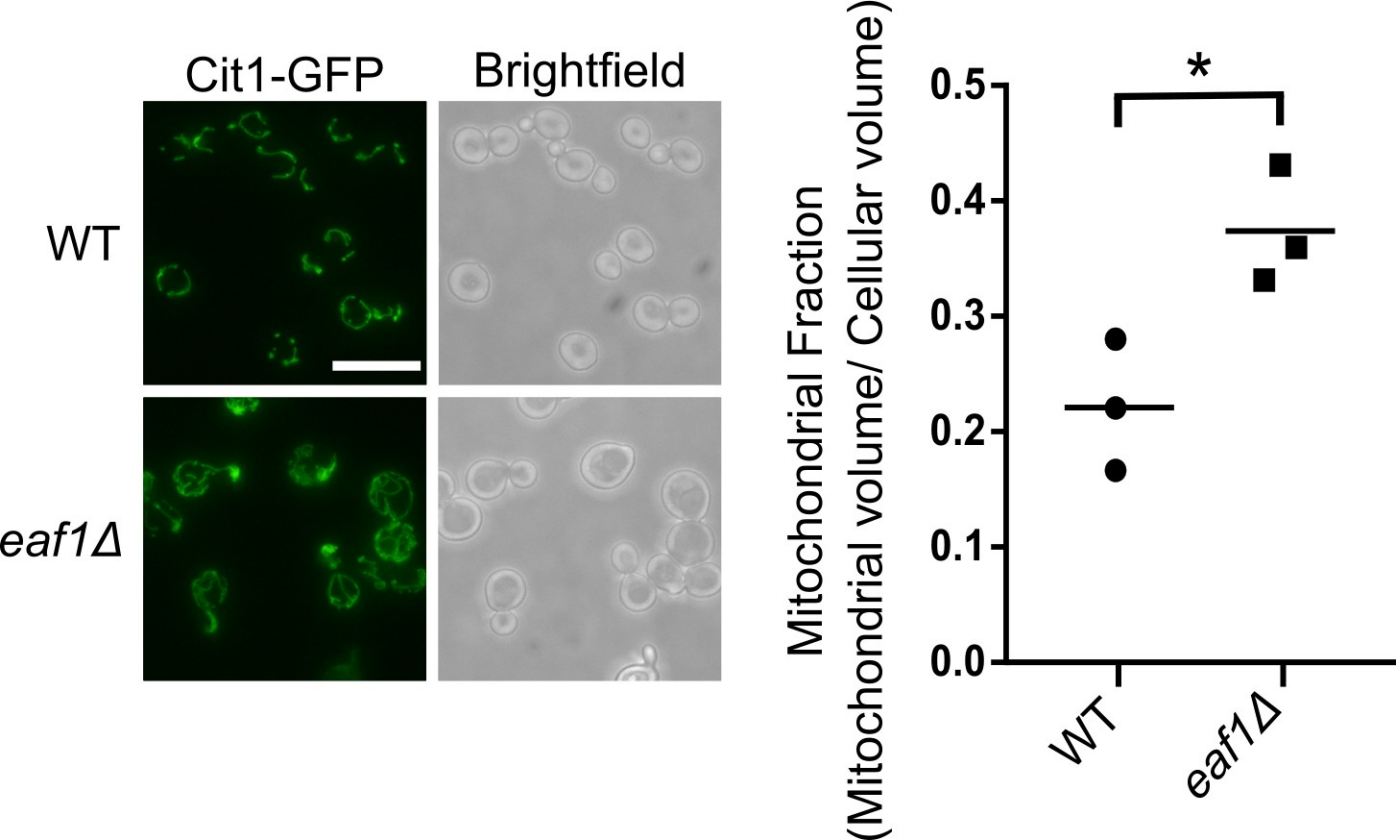
Mitochondrial morphology and volume are altered in NuA4 mutants. Cit1-GFP was used as a marker of the mitochondria to compare WT and *eaf1Δ* mitochondrial morphology by fluorescent microscopy. The mitochondrial volume was quantified based on the Cit1-GFP fluorescence using the MitoMap plugin for ImageJ which was then divided by the average total cellular volume of the strain to give the Mitochondrial Fraction (Raw cellular and mitochondrial volume measurements can be found in S7 and S8 Figs). Images are representative of 3 independent biological replicates and at least 50 cells per replicate were analyzed for quantification Scale bar = 10 μm. An unpaired T-test was run. * = p < 0.05. Horizontal bar in data represents the mean.

Our screen identified 14 mitochondrial proteins that had a change in GFP signal between WT and *eaf1Δ* (Figs 1A and S3). As all of the 14 proteins are resident mitochondrial proteins, rather than proteins that are relocalizing to the mitochondria, it suggests that mitochondrial elongation is occurring in *eaf1Δ* cells. To confirm this hypothesis we assessed 3 common markers of the mitochondria in both WT and *eaf1Δ* cells: Cit1-GFP, Aco2-GFP and MitoLoc [65], a plasmid expressing a mitochondrial localized GFP peptide (Figs 3 and S6). In each case, we detected hyperelongated mitochondria with increased branching in *eaf1Δ* cells. Despite the fact that *eaf1Δ* cells are almost 2x larger than WT cells (Figs 3 and S7), when the change in the mitochondrial volume is normalized to the increased cell size of the *eaf1Δ*, there is still a significant 1.7-fold increase in the mitochondrial volume relative to cell size (mitochondrial fraction) (Fig 3). When cell size is not accounted for, the mitochondrial volume of the *eaf1Δ* strain was found to be approximately three times larger than WT (S8 Fig). The mitochondrial morphology of the *esa1-ts* was also assessed after 2 hours at a semi-permissive temperature of 33°C and there was a slight change in mitochondrial morphology and but no significant change in mitochondrial fraction was identified (S9 Fig). The difference in the penetrance of the mitochondrial phenotype between the *eaf1Δ* and the *esa1-ts* may be explained by the difference in the mutation type. The *esa1-ts* is functional when grown at 25°C and the short 2-hour incubation at 33°C decreases NuA4 catalytic activity however extended temperature shift dramatically decreases viability. In contrast, deletion of *EAF1* disrupts the NuA4 complex, including substrate targeting, but the catalytic activity remains intact. It is also possible that Eaf1 plays a role outside of the NuA4 complex and Esa1 targeting, but that remains as a speculation, and the small effect of the *esa1-ts* on mitochondrial morphology seen in our experiments would suggest that this is not the case. Altogether, this indicates that a reduction in NuA4 activity results in altered mitochondrial morphology (a fused phenotype) and increased mitochondrial content per cell (mitochondriogenesis).

### While chaotic and elongated, the mitochondria in *eaf1Δ* cells are functional

**Fig 4:**
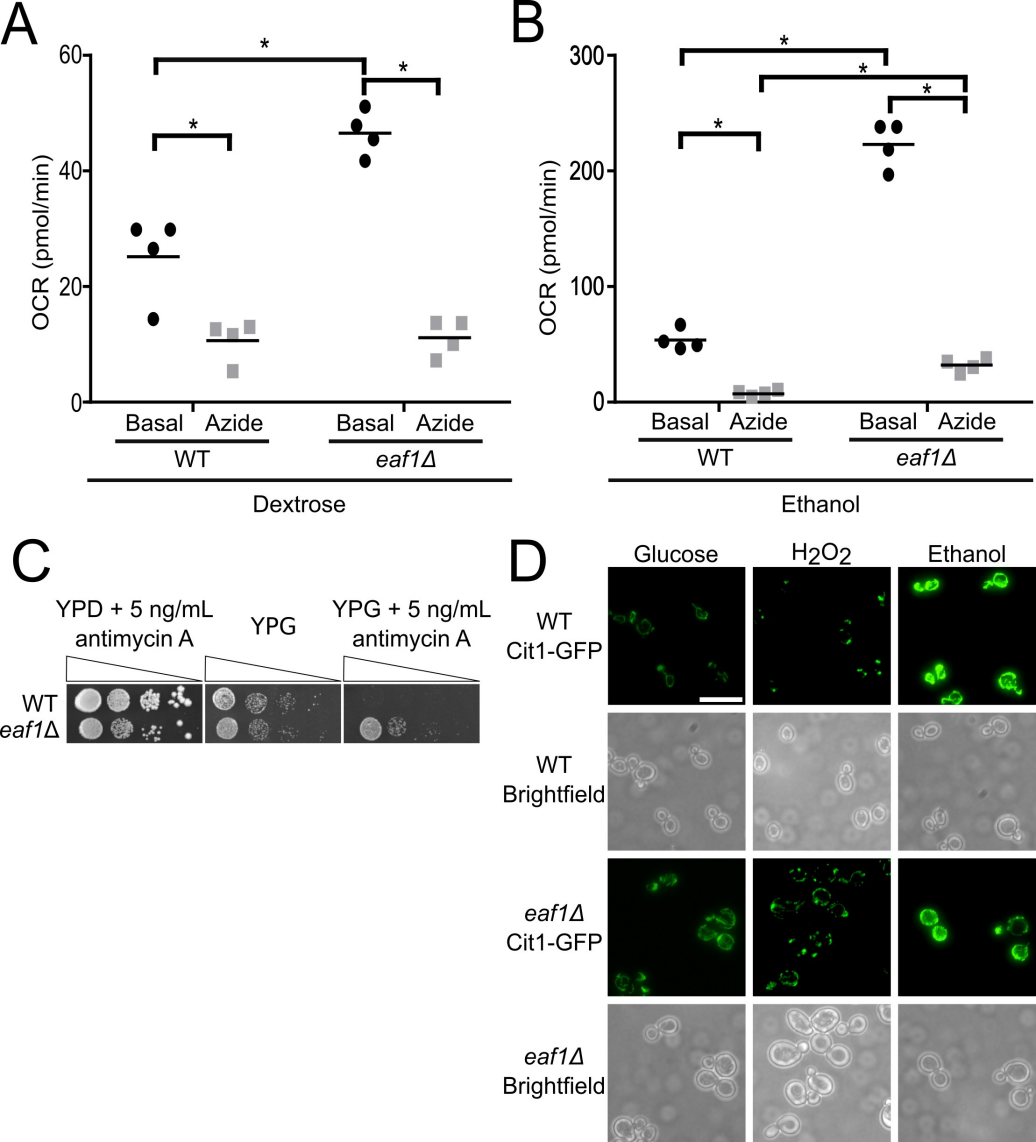
*eaf1Δ* mutants have functional mitochondria and are able to respond to biogenesis and fission signals. The oxygen consumption rate (OCR) of either WT or *eaf1Δ* cells was measured using the Seahorse Extracellular Flux Analyzer. After basal measurements, sodium azide was injected into the wells to shut off mitochondrial oxygen consumption and an additional set of measurements were taken. This was performed for (A) cells grown in dextrose media and (B) for cells grown in ethanol media for four biological replicates. ANOVA analysis was performed with a Tukey’s multiple comparison test comparing pairs of means. * = p < 0.05. Horizontal bars in the data represents the mean. (C) WT and *eaf1Δ* cells were serially diluted onto YP plates containing glucose (YPD) or the nonfermentable glycerol (YPG) in the presence of the mitochondrial complex III inhibitor antimycin A. (D) Mitochondrial responsiveness to environmental stressors was assessed by growing WT and *eaf1Δ* cells in the presence of ethanol to assess biogenesis or H_2_O_2_ to assess stress induced fission. Scale bar = 10 μm.

The structure of the mitochondria is important to its function [66]. Therefore, we sought to test if mitochondrial function was intact in *eaf1Δ* cells using three different assays. First, a Seahorse Extracellular Flux Analyzer (XF; Agilent) was used to measure mitochondrial bioenergetics. WT and *eaf1Δ* yeast were prepared overnight in YPD and then diluted in the morning into YPD or YPE (Ethanol), a non-fermentable carbon source that forces mitochondrial oxidative reactions over glycolysis. The oxygen consumption rate (OCR) of the mutant was compared to the control in YPD and YPE media and in both cases the *eaf1Δ* consumed significantly more oxygen than the WT yeast (Fig 4A and 4B). As a second measure of mitochondrial activity, we compared the viability of WT and *eaf1Δ* cells to treatment with a mitochondrial inhibitor. We anticipated that if *eaf1Δ* cells had increased functional mitochondria, they would be less sensitive to antimycin A, an inhibitor of the electron transport chain complex III. As expected, both WT and *eaf1Δ* cells are able to survive antimycin A treatments in the presence of glucose (YPD) when cells do not require mitochondrial function to survive. However, when grown on a non-fermentable carbon source such as glycerol (YPG) mitochondrial function is essential and WT cells were hyper-sensitive to antimycin A treatment compared to *eaf1Δ* cells (Fig 4C). Together with the mitochondrial bioenergetics assays, this suggests that not only do *eaf1Δ* cells have more mitochondria but that the mitochondria are functional. Finally, as the elongated mitochondria seen in *eaf1Δ* cells could reflect excessive mitochondrial fusion, or alternatively increased biogenesis, we next performed biogenesis and fragmentation assays. We first assessed the mitochondrial fission response upon exposure to hydrogen peroxide. Similar to WT cells, the mitochondria of *eaf1Δ* cells were able to fragment under hydrogen peroxide stress, indicating that mitochondrial fission is intact in *eaf1Δ* cells (Fig 4D). We then tested the ability of the *eaf1Δ* cells to undergo mitochondriogenesis in the non-fermentable condition of ethanol, where the mitochondria are required for ATP production and cell growth [67]. Both WT and *eaf1Δ* cells increased mitochondrial content upon ethanol treatment to a similar extent suggesting mitochondrial biogenesis is intact in *eaf1Δ* cells (Fig 4D). Consistent with this observation we determined that the increased mitochondrial volume of *eaf1Δ* cells is not reversed upon deletion of *HAP4*, the activator member of the Hap complex important for mitochondrial biogenesis (S10 Fig) [68]. Together our work shows that *eaf1Δ* cells not only have increased mitochondrial volume and are more fused and elongated, but that their mitochondria are functional and can respond to environmental stresses.

### Mitochondrial elongation in *eaf1Δ* cells can be partially reversed upon deletion of *BCY1*

**Fig 5:**
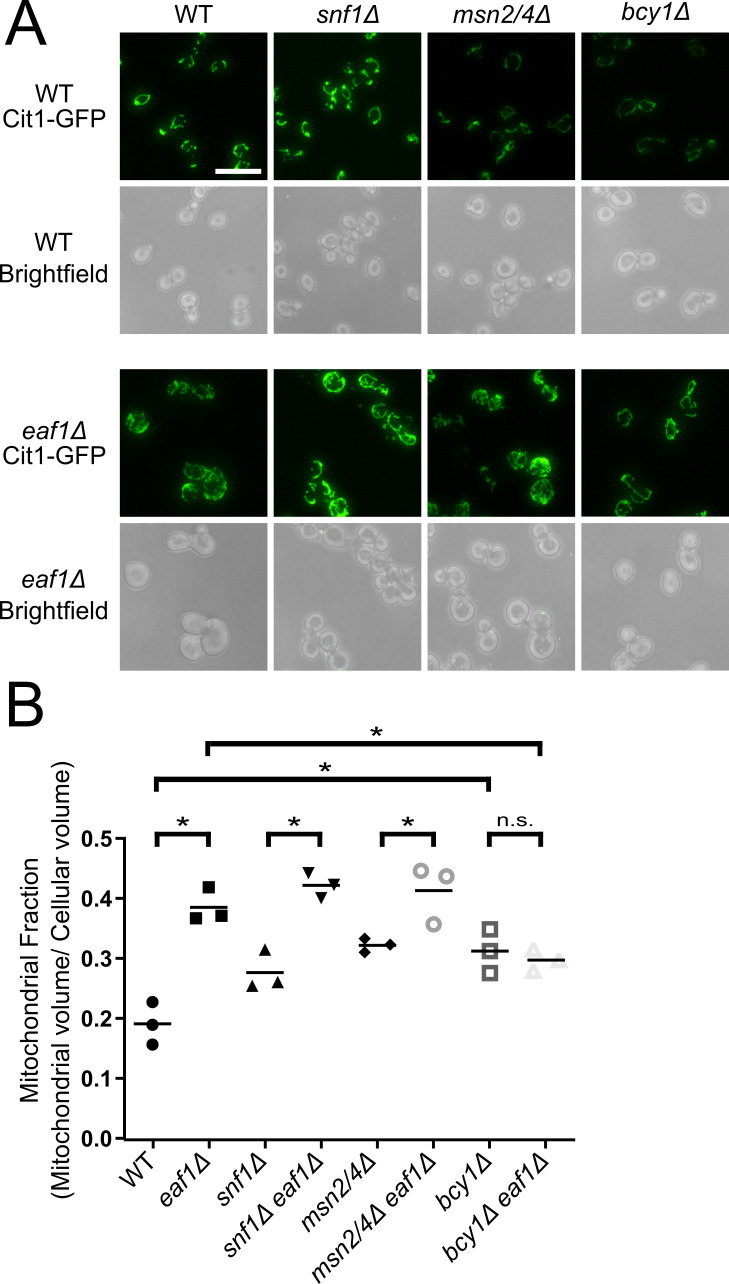
The increase in mitochondrial volume in an *eaf1Δ* is partially dependent on Bcy1. (A) Mitochondrial morphology was assessed in the indicated strains expressing the mitochondrial marker Cit1-GFP. Images are representative of three independent biological replicates. Scale bar = 10 μm. (B) The mitochondrial volume was quantified based on the Cit1-GFP fluorescence using the MitoMap plugin for ImageJ which was then divided by the average total cellular volume of the strain to give the Mitochondrial Fraction (Raw cellular and mitochondrial volume measurements can be found in S7 and S8 Figs). Images are representative of 3 independent biological replicates and at least 50 cells were quantitated per each biological replicate. ANOVA analysis was performed with a Tukey’s multiple comparison test comparing pairs of means. * = p<0.05, n.s. = p> 0.05, select relevant significance bars shown. Horizontal bars in the data represent the mean.

We next sought to determine a mechanism by which NuA4 may regulate mitochondrial morphology and volume. We first focused our attention on the AMPK/Snf1 and PKA-Msn2/Msn4 axis as they have both been implicated in mitochondrial dynamics [32,69–72]. Snf1 is a key kinase in yeast metabolic signalling [27]. In response to stress or low availability of glucose, and therefore high AMP, Snf1 kinase is activated and phosphorylates transcription factors leading to the transcription of glucose repressed genes is activated [27]. Snf1 has also been more directly associated with mitochondria by regulating fission, mitophagy, and mitochondrial biogenesis [26,28,73]. The transcription factors Msn2 and Msn4 are activators of glycolysis [46], and they have also been linked to autophagy and mitochondrial respiration [70,74]. Finally, PKA activity has been closely linked to the control of mitochondrial processes through multiple targets [75–81]. Therefore, we assessed the structure of the mitochondria using Cit1-GFP in WT and *eaf1Δ* backgrounds in combination with *snf1Δ*, *msn2msn4Δ*, and *bcy1Δ* (hyperactive PKA) (Fig 5). Though the morphology or mitochondrial fraction (mitochondrial volume divided by cellular volume) were not significantly changed in *snf1Δ* or *msn2Δmsn4Δ* cells, there was a slight increase in mitochondrial fraction in *bcy1Δ* cells, however the morphology of the mitochondria was similar to WT (Fig 5A). The elongated and branched structure and increased mitochondrial fraction in *eaf1Δ* cells was not impacted by the deletion of *SNF1* or *MSN2/4*. In contrast, mitochondrial structure in *bcy1Δ eaf1Δ* cells did not appear different from *bcy1Δ* cells (Fig 5A). Similarly, there was no significant increase in the mitochondrial fraction in *bcy1Δ eaf1Δ* cells compared to *bcy1Δ* cells (Figs 5B and S8). Our work suggests that NuA4 regulates mitochondrial morphology and volume through a mechanism dependent on the regulatory subunit of PKA, Bcy1, which is independent of Msn2/Msn4.

### Bcy1-GFP localization to the nucleus is dependent on NuA4

**Fig 6:**
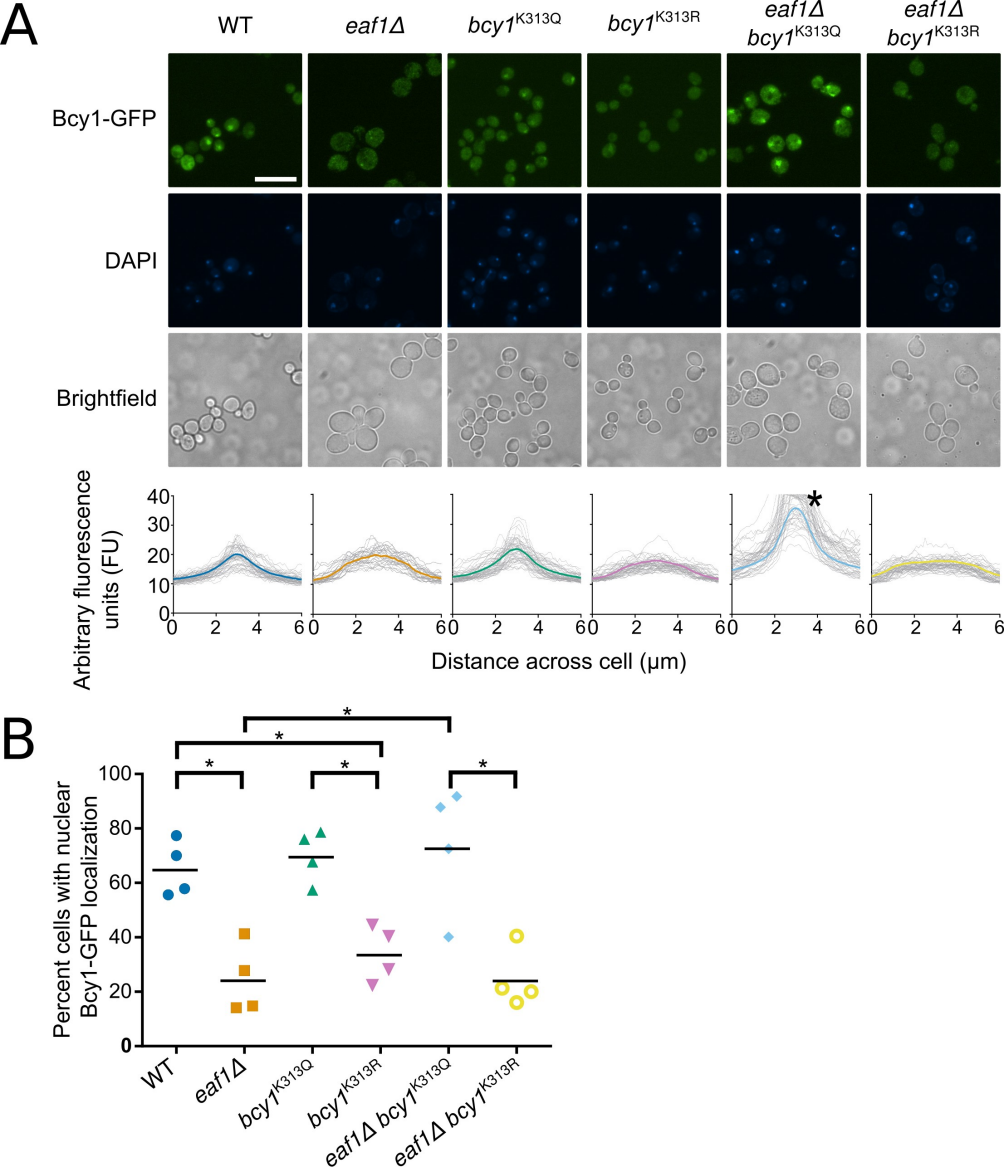
Bcy1 nuclear localization is dependent on NuA4 and the acetylation state of Bcy1-K313. (A) The localization of Bcy1-GFP, Bcy1^K313Q^-GFP and Bcy1^K313R^-GFP was assessed in WT and *eaf1Δ* yeast. Scale bar = 10 μm. Fluorescent intensity plots of Bcy1-GFP were derived by measuring the fluorescent intensity value of pixels across a nucleus-centered line using Plot Profile in ImageJ Software (Lower panel). The coloured line is the mean of three biological replicates for each strain, and profiles for 50 cells are shown in dotted lines. * For *eaf1*Δ *bcy1*^*K313Q*^ the y-axis has been cropped at 40 for comparison. The height of the peak corresponds with the pixel intensity maximum of the nucleus and therefore approximates the abundance of Bcy1-GFP in the nucleus. (B) The percentage of cells with nuclear localization of Bcy1-GFP was calculated for each strain indicated. A minimum of 50 cells were counted for each of the four biological replicates. ANOVA analysis was performed with a Tukey’s multiple comparison test comparing pairs of means. * = p<0.05, relevant significance bars shown. Horizontal bars in the data represent the mean.

The glycogen synthesis and mitochondrial content phenotypes identified in the screen are partially, if not fully, dependent on Bcy1, the regulatory subunit of yeast PKA (catalytic subunits are Tpk1/2/3). Additionally, the deletion of *BCY1* appears to reverse the growth defect of an *eaf1Δ* (Fig 2B). Interestingly, Bcy1 was also one of the 23 hits from our phenomic screen. While our screen and other studies [34,41] have determined that Bcy1-GFP is enriched in the nucleus in log phase WT cells grown in glucose, upon the deletion of *EAF1* in the same conditions, Bcy1-GFP nuclear signal decreases with an increase in cytoplasmic localization (Figs 1B, 6A and 6B). Fluorescent intensity profile plots of Bcy1-GFP signal across the whole cell centred on the nucleus indicate that within the population there is an accumulation of Bcy1-GFP in the nucleus of WT cells, however this signal is spread more evenly across *eaf1Δ* cells indicative of dispersion of Bcy1-GFP throughout the cell (Fig 6A). The proportion of cells with prominent nuclear localization of Bcy1-GFP was also quantified demonstrating that nuclear localization of Bcy1-GFP is reduced in an *eaf1Δ* (Fig 6B). While the localization of at least Tpk1 has been suggested to be associated with Bcy1 localization [34,41], we did not find any associated changes in the localization of Tpk1, 2, or 3 upon deletion of *EAF1* in our screen or in follow up analysis (S11 Fig).

Previous work has suggested that Bcy1 binding to the catalytic subunits of PKA may be regulated through NuA4-dependent acetylation of lysine K313 [31]. It has been proposed that the unacetylated form of Bcy1 can interact with and inhibit PKA subunits, Tpk1/2/3, but when Bcy1 is acetylated by NuA4 the Bcy1-TPK interaction is disrupted leading to activation of PKA. Our work suggests that NuA4 is also regulating Bcy1 localization. Hence, we sought to establish if the acetylation state of Bcy1-K313 affects its localization. We used CRISPR-Cas9 to create the Bcy1^K313Q^-GFP and Bcy1^K313R^-GFP mutations in the yeast genome to assess the impact of K313 lysine acetylation on the localization of Bcy1. The structure and electron distribution of arginine (R) is a good resemblance for a lysine residue in the unacetylated state while glutamine (Q) resembles the acetylated form of lysine [82]. We first assessed the nuclear localization of these mutations using DAPI stain as a marker for the nucleus and quantified cells with an enrichment of Bcy1-GFP in the nucleus. The nuclear enrichment is maintained in the Bcy1^K313Q^-GFP acetylated mimic mutant but not in the Bcy1^K313R^-GFP unacetylated mimic (Fig 6). This suggests that mislocalization of Bcy1-GFP in *eaf1Δ* cells is potentially due to a lack of acetylation on Bcy1-K313. If this hypothesis is correct, we would anticipate that Bcy1^K313Q^-GFP expressed in *eaf1Δ* cells would restore localization to the nucleus. As expected, the Bcy1^K313Q^-GFP expressed in *eaf1Δ* background is enriched in the nucleus while the non-acetylatable lysine mimic Bcy1^K313R^-GFP expressed in *eaf1Δ* is dispersed across the cell (Fig 6A and 6B). Together our results suggest that the Eaf1-dependent localization of Bcy1 is regulated by the acetylation state of K313.

### Bcy1-K313 mutation affects mitochondrial morphology and glycogen content

**Fig 7:**
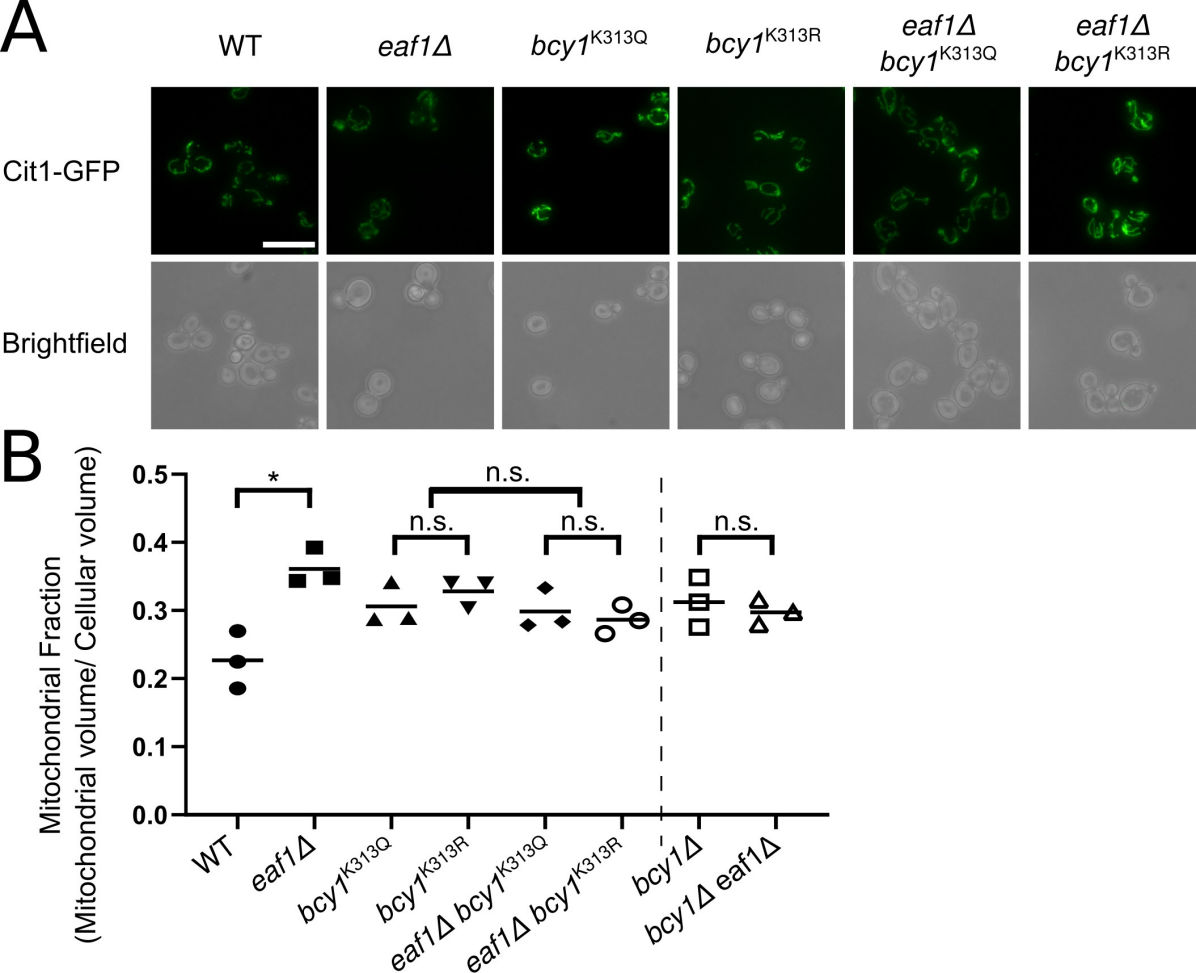
Assessment of mitochondrial volume in Bcy1-K313Q/R mutants. **(**A) The mitochondrial structure and volume were assessed in the indicated strains expressing the mitochondrial marker Cit1-GFP. Scale bar = 10 μm. (B) The mitochondrial volume was quantified based on the Cit1-GFP fluorescence using the MitoMap plugin for ImageJ which was then divided by the average total cellular volume of the strain to give the Mitochondrial Fraction (Raw cellular and mitochondrial volume measurements can be found in S7 and S8 Figs). Images are representative of 3 independent biological replicates and at least 50 cells per replicate were analyzed for quantification. This volume was compared to previously calculated *bcy1Δ* and *bcy1Δ eaf1Δ* mitochondrial volume measurements (x-axis break represents experiments previously shown in Fig 5). ANOVA analysis was performed with a Tukey’s multiple comparison test comparing pairs of means. * = p<0.05, n.s. = p>0.05, relevant significance bars shown. Horizontal bars in the data represent the mean.

We next asked whether the Bcy1-K313 dependent change in localization of Bcy1 in *eaf1Δ* cells is contributing to either the increase in glycogen or mitochondrial content. We first assessed mitochondrial morphology and determined that there is slight but not significant increase in the mitochondrial fraction and mitochondrial elongation in both the *bcy1*^*K313R*^ and *bcy1*^*K313Q*^ mutants similar to that of *bcy1*Δ mitochondria (Figs 7A, 7B and S8). Further, both the *bcy1*^*K313Q*^ and *bcy1*^*K313R*^ could both slightly suppress the increase in the mitochondrial fraction and the mitochondrial elongation in *eaf1Δ* cells (Figs 7A, 7B and S8) a phenotype similar to the mitochondria of the *bcy1Δ eaf1Δ* (Figs 5 and 7). As mitochondrial morphology is not significantly different in *bcy1*^*K313R*^ and *bcy1*^*K313Q*^ mutants, it suggests that K313 is important for Bcy1 function and is not necessarily acetylation state specific. Therefore, there may also be an additional level of Bcy1 regulation by NuA4 outside of direct K313 acetylation.

**Fig 8:**
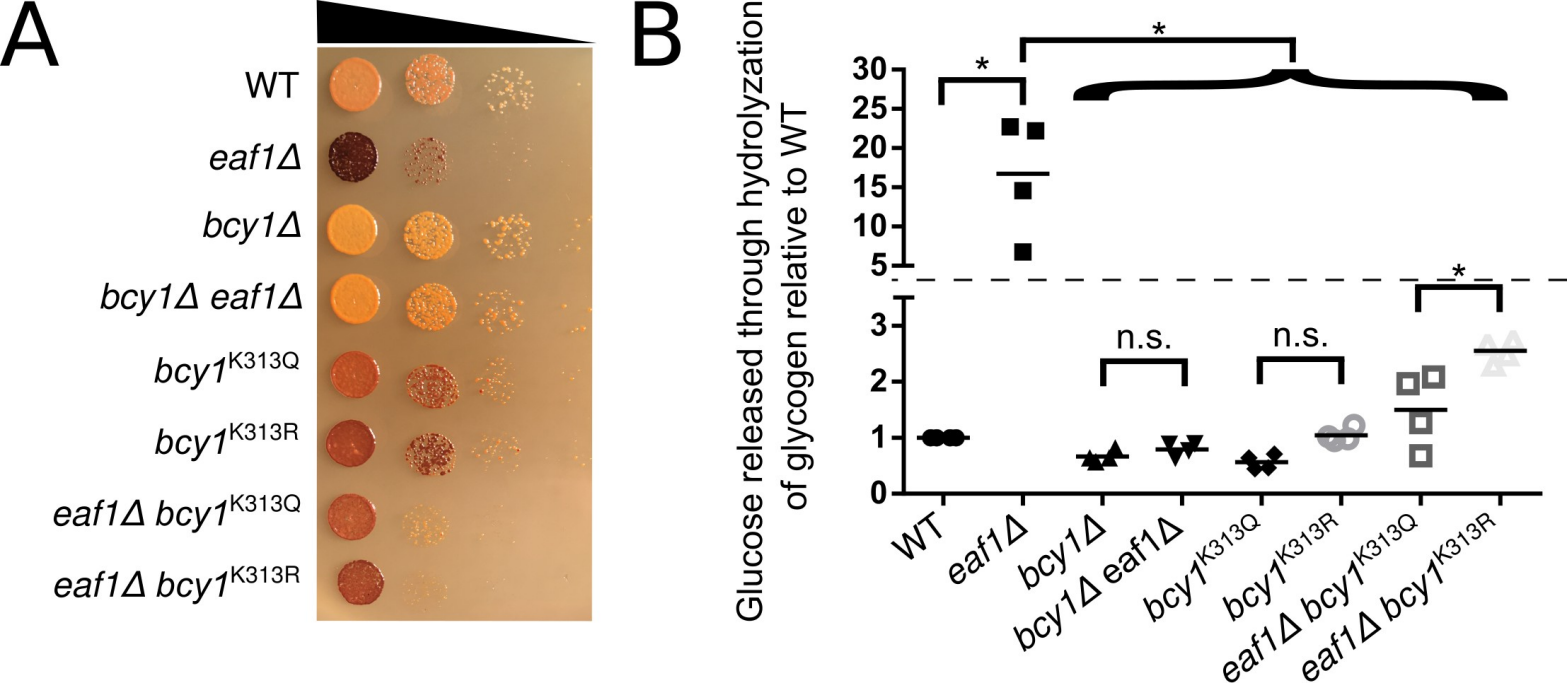
Bcy1-K313Q/R mutants partially suppress glycogen accumulation of *eaf1Δ* cells. **(**A) Cellular glycogen levels was assessed using iodine staining. The indicated strains were serially diluted on to YPD plates, grown at 30°C for 24 hours prior to exposure to iodine crystals. Darker staining represents more glycogen within the cell. Image is representative of three independent dot assays. (B) Cellular glycogen content was assessed by measuring glucose released from hydrolyzation of glycogen. Cellular glycogen was extracted and treated with amyloglucosidase to break down glycogen into glucose. Glucose was then measured directly using the Glucose Colorimetric/Fluorometric assay kit (Biovision, K606). The amount of glucose released through the hydrolyzation of glycogen in each replicate was normalized to that of the WT strain. The Y-axis is broken (horizontal dotted line) in order to best show the data. ANOVA analysis was performed with a Tukey’s multiple comparison test comparing pairs of means. * = p<0.05, n.s. = p>0.05, relevant significance bars shown. Horizontal bars in the data represents the mean.

To assess the impact of the *bcy1* mutants on glycogen, we performed both iodine staining of cells and measured glucose released from the hydrolyzed cellular glycogen [64,83]. As expected, the *bcy1*^*K313R*^ cells displayed higher glycogen levels (darker iodine staining) than WT, *bcy1*^*K313Q*^, and *bcy1Δ* cells, yet lower than the *eaf1Δ* cells (Fig 8A). However, *bcy1*^*K313Q*^ cells were also stained darker compared to WT cells, suggesting glycogen levels are increased in both mutants. This may be the result of the inability of a glutamine residue to perform all functionality of an acetylated lysine. The intermediate effects of the *bcy1* mutants were also seen in the suppression of glycogen accumulation in *eaf1Δ* cells. The *bcy1*^*K313Q*^ *eaf1*Δ appeared consistently slightly lighter in iodine staining that *bcy1*^*K313R*^ *eaf1*Δ, however the *bcy1*^*K313Q*^ mutant does not fully suppress glycogen accumulation in *eaf1*Δ cells (Fig 8A) [31]. Similar trends were detected by hydrolyzation of cellular glycogen. There was approximately a 15-fold increase in glucose released from *eaf1Δ* cells relative to WT cells (Fig 8B). This increase was suppressed by the deletion of *BCY1* in the *eaf1Δ* strain. Consistent with the iodine staining, the glucose released from all of the Bcy1-K313 mutants was closer to the WT cells than the *eaf1Δ* cells. However, while not significant there was a small increase in the glucose released by hydrolyzation in *bcy1*^*K313R*^ cells compared to the *bcy1*^*K313Q*^ cells and the *eaf1Δ bcy1*^*K313R*^ cells did show a significant increase in glucose release relative to the *eaf1Δ bcy1*^*K313Q*^ cells. This suggests that the accumulation of glycogen in *eaf1*Δ cells is only partially due to decreased acetylation on K313 and mislocalization of Bcy1. Together this suggests that while acetylation state of Bcy1-K313 may regulate its subcellular localization, the acetylation of this target site alone is not fully responsible for NuA4-dependent regulation of mitochondrial morphology or PKA-regulation of glycogen biosynthesis.

### PKA phosphorylation substrates are impacted by deletion of *EAF1*

**Fig 9:**
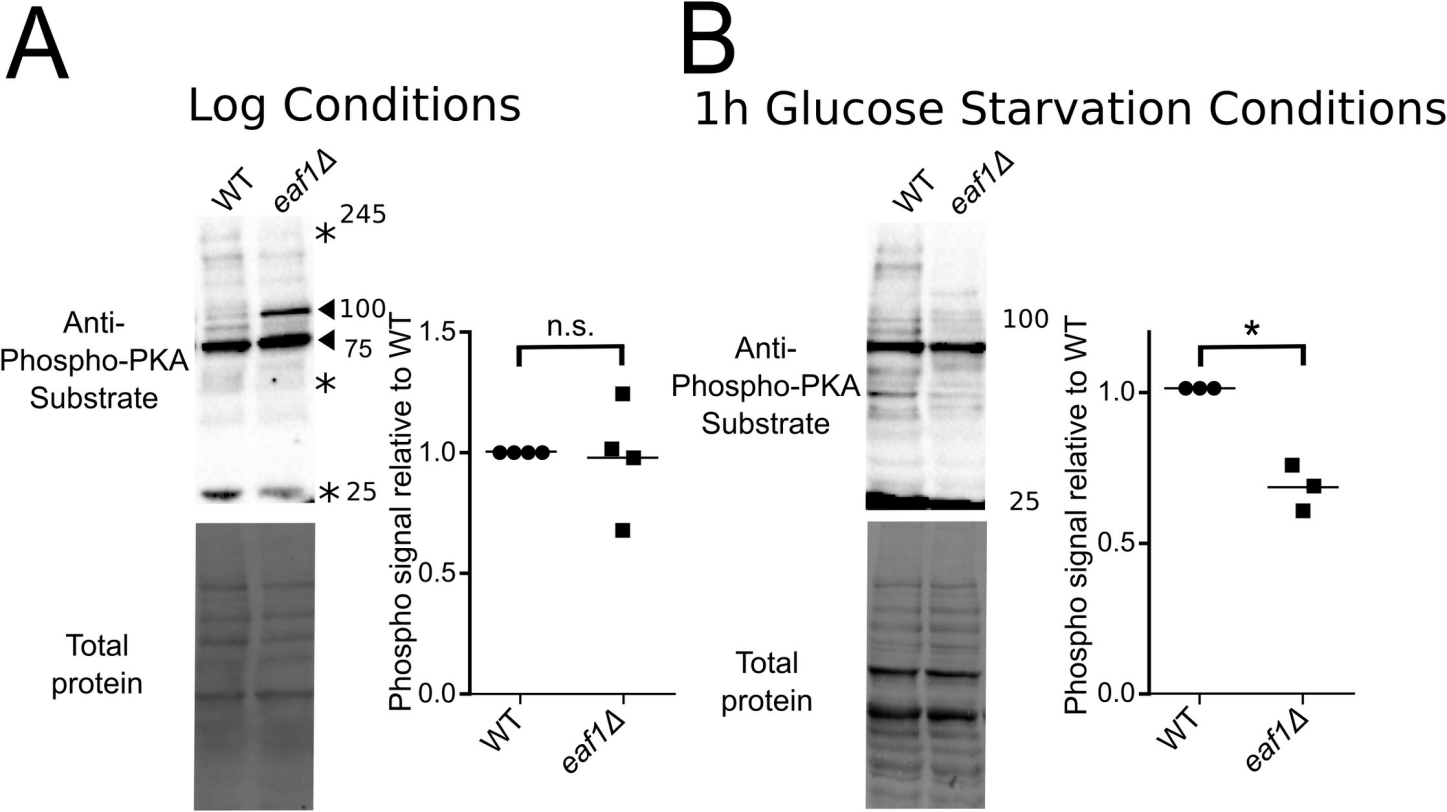
PKA activity is altered in an *eaf1Δ*. (A) PKA substrate phosphorylation under glucose rich conditions was assessed by quantitative western blot analysis using whole cell extracts from WT and *eaf1Δ* strains. Panel to the left is a representative of a total phosphorylation and a total protein blot (Stars highlight bands that decreased and arrows highlight bands that increase in *eaf1Δ* extracts); panel to the right is the quantification of western blots for four independent biological replicates where the total phosphorylation signal intensity was normalized to total protein. (B) PKA substrate phosphorylation under glucose starvation conditions was assessed by quantitative western blot analysis using whole cell extracts from WT and *eaf1Δ* strains that were starved for glucose for 1 hour. Panel to the left is a representative total phosphorylation and total protein blot; panel to the right is the quantification of western blots for three independent biological replicates where the total phosphorylation signal intensity was normalized to total protein.

Though mutations that mimic the acetylated and un-acetylated state of Bcy1-K313 impact its interaction with Tpk1/2/3 [31] and its subcellular distribution (Fig 6), our work suggests NuA4- regulation of PKA activity maybe more complex then simply acting as an on or off switch through Bcy1 acetylation. Therefore, we next asked if deletion of *EAF1* affects global PKA activity or if only a subset of PKA substrates are impacted. Overall activity of the yeast PKA catalytic subunits (Tpks) was assessed by blotting whole cell extracts from WT and *eaf1Δ* cells with a Phospho-PKA Substrate (RRXS*/T*) antibody, which is commonly used to assess PKA activity [84–86]. Interestingly, in glucose-rich conditions there was no significant difference in total phosphorylation between WT and *eaf1Δ* cells, but there are reproducible distinct changes in substrates detected by the anti-phospho-PKA antibody (Fig 9A). One protein at approximately 100 kDa reproducibly appeared as an increased phosphorylation target in the *eaf1Δ* strains while a protein at 25 kDa appeared to have slightly reduced phosphorylation (Fig 9A). As PKA activity is affected by glucose abundance, we also assessed phosphorylation of PKA targets in the WT and *eaf1Δ* cells after 1 hour of glucose starvation. Under glucose starvation the *eaf1Δ* cells had approximately a 30% reduction in total phosphorylation compared to WT cells, including complete loss of some phospho-PKA substrates (Fig 9B). These results suggest that NuA4 is not simply turning on or off PKA activity but is playing a potential role in regulating PKA substrates.

## Discussion

We found that 23 metabolic proteins change localization and/or abundance upon disruption of the NuA4 complex (Figs 1 and S3). Remarkably, we determined that the majority of the detected changes, including induction of the glycogen biosynthesis pathway and mitochondrial elongation, were at least partially dependent on NuA4-target Bcy1 (Figs 2 and 5). Given the well-established role of Bcy1 in regulating the PKA catalytic subunits (Tpk1/2/3), this suggests that many of the cellular impacts of NuA4 on metabolism are likely mediated through regulation of PKA.

**Fig 10:**
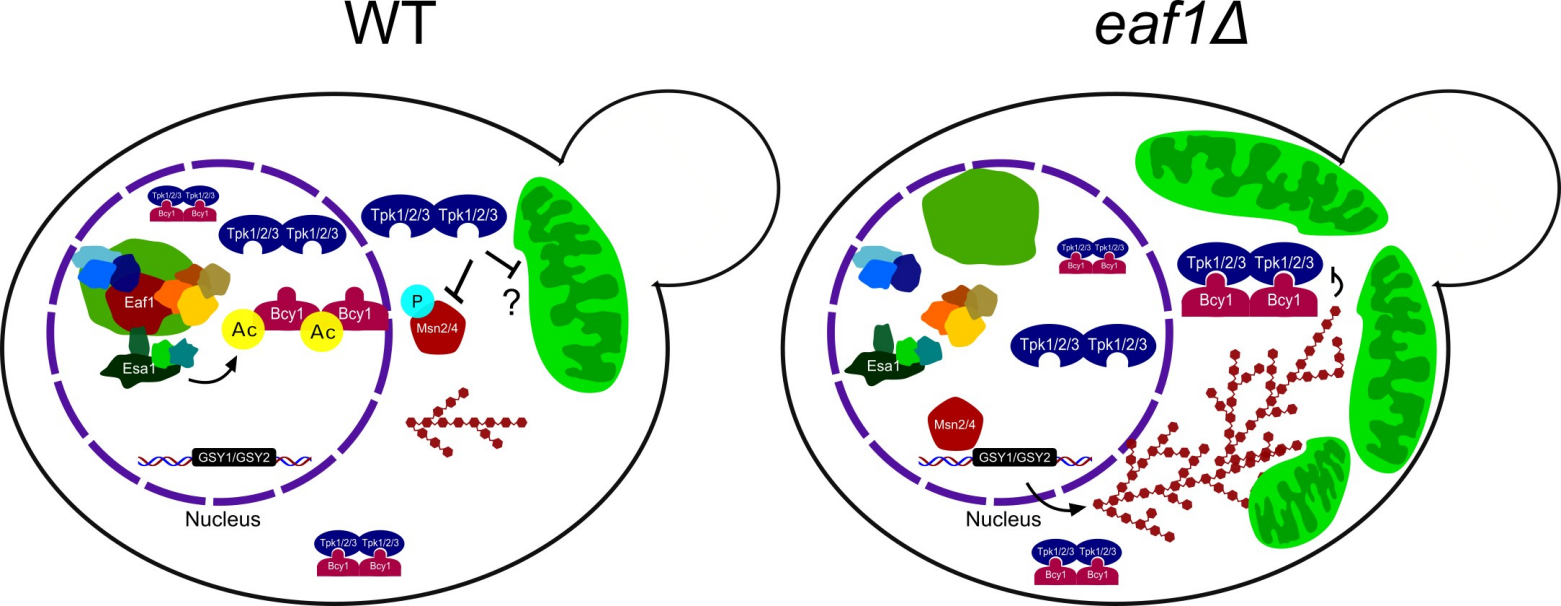
Model for regulation of mitochondrial morphology and glycogen synthesis by NuA4. We propose that NuA4 regulates Bcy1 acetylation and subcellular distribution leading to a change in Tpk activity downstream, thereby affecting regulation of mitochondrial volume and glycogen synthesis gene transcription.

How is NuA4 impacting PKA activity? Contrary to many other metabolic enzymes where acetylation is acting as an “on” or “off” switch, our work suggests that NuA4-dependent regulation of PKA is more subtle. Our work suggest a model that in the absence of *EAF1* or NuA4 activity, acetylation of Bcy1-K313 is decreased allowing for Bcy1 to translocate to the cytoplasm altering the inhibition of some or all of the yeast PKA enzymes, Tpk1/2/3 (Fig 10). Indeed, upon deletion of *EAF1*, under glucose rich and replete conditions, we demonstrated that substrates detected by the phospho-PKA substrate antibody are not simply globally decreased, but rather altered.

### NuA4 regulation of glycogen synthesis

Our screen determined that glycogen synthesis pathway proteins are increased in *eaf1Δ cells*, leading to increased glycogen content (Figs 2B and 8). The small differences between the glycogen content results of the iodine staining and hydrolyzation by amyloglucosidase may be accounted for by difference in the chemistry of these methods [83]. Iodine binds to endogenous glycogen to produce the red/brown colour in the cell while glycogen must be extracted for hydrolyzation then glycogen is indirectly measured through the amount of glucose released [83]. Each have limitations, but together they strongly support that *eaf1Δ* cells have significantly more glycogen than WT. This fits well with the literature demonstrating that *GSY1*, *GSY2*, and *GDB1* are established transcriptional targets of Msn2/Msn4 [46,59,60] and that other Msn2/Msn4-dependent genes are derepressed in NuA4 mutants [20,87]. As the deletion of *EAF1* results in the reduction of nuclear Bcy1-GFP, enriched nuclear Msn2-GFP, and an induction of Gsy2-GFP, a simple explanation is that NuA4 is regulating PKA activity simply through regulation of Bcy1 localization. However, this is not the case as *bcy1*^*K313R*^ cells do not accumulate glycogen to similar levels of *eaf1Δ* cells (Fig 8), despite the fact that Bcy1^K313R^-GFP has a similar decrease in nuclear enrichment as Bcy1-GFP in *eaf1Δ* cells (Fig 6). Even more surprisingly, although the *eaf1Δ bcy1*^*K313R*^ cells have more glycogen than the *eaf1Δ bcy1*^*K313Q*^ cells, both have far less glycogen accumulation than the *eaf1Δ* alone. This suggests that NuA4 may also regulate the PKA-Msn2/Msn4 axis through additional mechanisms outside of Bcy1-K313 acetylation.

### NuA4 regulation of mitochondrial morphology

How is deletion of *EAF1* resulting in elongation of the mitochondria? A simple explanation could have been that NuA4 regulates transcription of genes required for mitochondrial biogenesis and fusion. However, we determined that Eaf1-dependent mitochondrial changes are not mediated by Msn2/4 (Fig 5) or Hap4 (S10 Fig). Indeed, transcriptome studies do not indicate any up-regulation of genes involved in mitochondrial biogenesis, fusion, or fission upon deletion of *EAF1* [18], suggesting NuA4’s impact on mitochondrial morphology is not due to transcriptional defects.

We found that the mitochondrial defects in *eaf1*Δ cells can be partially rescued by deletion of *BCY1* (Fig 5), which suggests that altered PKA activity in NuA4 mutants contributes to mitochondrial elongation. However, deletion of *BCY1* or mutation of Bcy1-K313 to either R or Q resulted in increases in the mitochondrial fraction per cell, albeit not as high as seen in *eaf1Δ* cells (Figs 5 and 7). This suggests that both K313 mutants have compromised Bcy1 function that results in increased mitochondrial function.

At first, this seems counter-intuitive. How could *bcy1*Δ cells with increased PKA activity and *eaf1Δ* cells that we show have altered PKA activity and many hallmarks of decreased PKA activity, both lead to increased mitochondrial content? Our work suggests that NuA4 and acetylation of Bcy1 is not simply acting as an on or off switch for PKA activity. Rather, we show that under glucose rich conditions, deletion of *EAF1*, and presumably the collapse of NuA4, alters the PKA-substrate profile, with some phospho-PKA substrates increasing while others decreasing (Fig 9). Though one can not rule out the possibility that deletion of *EAF1* is impacting the protein levels of PKA substrates, indeed our high content screen does detect changes in protein levels (Figs 1 and S2), overall the impact of *EAF1* on global transcription is minor [18]. Future studies will be needed to identify the extent by which NuA4 impacts PKA activity and substrate selection, including through acetylation of Bcy1 or other mechanisms.

Interestingly, PKA has been extensively linked to various aspects of mitochondrial regulation, including biogenesis and disassembly. PKA activity has been generally demonstrated to promote mitochondrial fusion and function, explaining why the deletion of *BCY1* alone may result in slightly more mitochondria than in WT cells [35]. Studies have shown that PKA phosphorylates Drp1, a key mitochondrial fission protein, inhibiting its function and promoting fusion [88,89]. Similarly, PKA phosphorylates MIC60 reducing PINK1 presence at the mitochondria, preventing mitochondrial degradation [90]. Additionally, deleting *BCY1* and high cAMP conditions, both of which should increase PKA activity, each led to an increase in mitochondrial content in yeast, while deletion of *TPK3* (one of the yeast PKA catalytic subunits) was associated with a decrease in cytochrome C content [32,91]. Outside of overall mitochondrial structure and content regulation PKA also directly phosphorylates members of the electron transport chain as well as translocase of the outer membrane (TOM) proteins responsible for mitochondrial protein import [78–80]. Finally, PKA activity has been associated with mitochondriogenesis through phosphorylation and activation of CREB leading to transcription of PGC1α, a key cotranslational activator of mitochondriogenesis [40,92].

Given this research, it is clear that NuA4 is regulating mitochondrial elongation through PKA, but the mechanism(s) remain to be established. There are complex nuances associated with PKA signalling, and perhaps it is not as cut and dry as increased versus decreased PKA activity affecting mitochondrial regulation. For example, PKA dependent phosphorylation of two different apoptotic proteins Bad and Bim has opposite functions, preventing and promoting apoptosis respectively [93–95]. While we did not see large scale changes in the localization of any of the Tpk subunits in an *eaf1Δ* when compared to WT (S11 Fig), we cannot rule out that NuA4 is modulating distinct pools of PKA (e.g. Tpk1 vs Tpk2, or different compartmentalized groups) that have different effects on mitochondrial biology. Indeed, due to functional redundancies, the biological roles of individual PKA subunits in yeast have yet to be discerned in detail.

### NuA4 dependent regulation of Bcy1 subcellular localization

Previous high throughput protein-fragment complementation assay (PCA) studies identified a role for NuA4 in regulating PKA [31]. In particular, this study determined that upon deletion of *EAF1* or *EAF7* the Tpk1-Bcy1 (PKA1) interaction is increased under both glucose and galactose conditions, however Tpk2-Bcy1 (PKA2) was not impacted, supporting the idea that NuA4 may differentially regulate the individual PKA subunits. Further, through PCA-based assay in mammalian cells they showed that increasing acetylation through either overexpression of KATs or inhibition of KDACs results in a reduction of PKA type II interactions. Though not directly tested, it was proposed that acetylation of Bcy1-K313 reduced its interaction with Tpk1, hence relieving inhibition of PKA. While this may be the case, our work shows that Eaf1 (and presumably NuA4) and acetylation are regulating the subcellular localization of Bcy1. Bcy1-GFP is predominantly nuclear in glucose grown cells [34] while in the absence of *EAF1*, Bcy1-GFP is present in both nuclear and cytoplasmic compartments (Figs 1 and 6). Further, Bcy1^K313R^-GFP, which mimics the unacetylated state, displays a similar disperse cellular signal as seen for Bcy1-GFP in *eaf1Δ* cells, and the Bcy1^K313Q^ -GFP mutant maintains a nuclear localization even when *EAF1* is deleted (Fig 6).

The subcellular localization of Bcy1 is extremely important as the compartmentalization of PKA components plays a role in regulating its activity [42]. For example, in the mammalian system, specific A-Kinase anchor proteins (AKAPs) have evolved to localize PKA to different areas of the cell [42,96]. While mammalian AKAP homologs have not been identified in yeast, the localization of yeast PKA members is dependent on carbon source availability and environmental stresses and many interacting proteins have been identified [34,42,97]. In glucose, Bcy1 is predominantly nuclear [34,42], however upon glucose starvation or when grown in non-fermentable Ethanol or Glycerol media, Bcy1 has a nucleo-cytoplasmic distribution [34,42]. In contrast, each TPK subunit has distinct subcellular distribution, while Tpk2 is predominantly nuclear, Tpk1 and Tpk3 are evenly distributed between nucleus and cytoplasm under logarithmic growth in glucose. Interestingly, when cells enter stationary phase, while Tpk1 and Bcy1 are distributed across the cytoplasm and nucleus, Tpk2 and Tpk3 are enriched in P-bodies that are largely devoid of Bcy1 [42]. In the absence of nuclear localized Bcy1, others have shown that nuclear Tpk1 is also reduced, suggesting Bcy1 may not only regulate PKA activity but sequester PKA catalytic subunits to specific locations [34]. Interestingly, while we determined that upon deletion of *EAF1*, Bcy1-GFP nuclear localization is reduced (Figs 1 and 6), we did not see a change in Tpk1-GFP subcellular distribution (S11 Fig). The fact that Tpk1 localization is not dramatically altered in an *eaf1Δ* cell suggests that enough Bcy1 remains in the nucleus to maintain pools of Tpk1 in the nucleus. Alternatively, while our screen and direct analysis did not detect any dramatic changes in the localization or abundance of the Tpks, we cannot rule out if subtle changes in Tpk1, Tpk2, and Tpk3 subcellular localization are regulated by NuA4 through Bcy1-K313 acetylation or other means. Further exploration will require detailed temporal and spatial analysis of subcellular localization and activity of individual PKA subunits upon modulation of NuA4 activity.

As the disperse localization of Bcy1-GFP in *eaf1Δ* cells grown in glucose conditions (Fig 6) looks similar to localization of Bcy1-GFP upon glucose deprivation [34,41], one simple explanation is that *eaf1Δ* cells are defective for glucose sensing resulting in the mislocalization of Bcy1-GFP indirectly. However, our work suggests that subcellular localization is dependent on the acetylation state of Bcy1-K313 (Fig 6), which suggests NuA4 is directly regulating Bcy1 localization through acetylation. Presently it is not known if NuA4 activity changes upon starvation or glucose deprivation, however NuA4 is implicated in regulating stress granule formation upon glucose deprivation [51] and glucose-starvation apoptosis in cancer cells [98]. Therefore, it will be interesting to determine if the acetylation state of Bcy1 changes upon glucose deprivation and if so, if this is dependent on NuA4.

Regulation of Bcy1 localization by post-translational modification is not new. The Yak1-dependent phosphorylation of the N-terminal domain of Bcy1 drives its localization to the cytoplasm where it is retained by Zds1 [34,41]. Interestingly the Bcy1-K313 site is not within the established N-terminal clusters required for interaction with Zds1 and cytoplasmic localization of Bcy1. This suggest that other proteins maybe interacting with the acetylated or unacetylated versions of Bcy1 aiding in changing its subcellular distributions. Though we or others have yet to establish if acetylation of Bcy1 is also impacting the physical interaction with TPKs, what is clear is that deleting *EAF1* decreases Bcy1 nuclear localization and results in phenotypes that suggest PKA activity is decreased, including increased glycogen production, Msn2 relocalization to the nucleus, and mitochondriogenesis (Figs 1, 2, and 7). However, localization of Bcy1 is clearly not the only contributor of NuA4-dependent regulation of PKA activity, as *bcy1*^*K313R*^ and *bcy1*^*K313Q*^ mutants, despite having strong separation of Bcy1 localization (Fig 6), have similar impacts on mitochondria morphology and only small differential impacts on glycogen accumulation (Figs 7 and 8). One possibility that should be considered is that while K to R and K to Q are used as mimics for acetylation states *in vivo*, these are in fact not ideal. For example, NuA4 has been shown to regulate the oxysterol binding protein Kes1 *in vivo*, but the Kes1 acetylated and unacetylated mimics proteins do not fully replicate Kes1 acetylated and unacetylated proteins *in vitro* biochemical assays [48]. The limitation of these acetylation state mimics may explain why the *bcy1*^*K313R*^ and *bcy1*^*K313Q*^ mutants appear to act similarly to each other in some cases. However, their additional similarity to *bcy1*Δ and differences from WT and *eaf1Δ* in terms of mitochondrial morphology and glycogen content does support an important function of the K3l3 position of Bcy1, although how NuA4 dependent acetylation of this residue is involved awaits further study.

## Conclusions

Our results clearly demonstrate that NuA4 is regulating the localization of a master regulator of cellular metabolism, Bcy1, the regulatory subunit PKA. Additionally, both the increased glycogen synthesis proteins and mitochondrial elongation of *eaf1*Δ cells identified in our screen are dependent on Bcy1, and PKA activity is altered upon deletion of *EAF1*, supporting our proposed PKA regulation. Finally, our screen suggests that NuA4 may be regulating multiple metabolic pathways through the localization of proteins. Taken together, our work indicates that human homolog of NuA4, Tip60, may play a key role in controlling cellular metabolism, perhaps explaining how aberrant Tip60 function may be important in the development of human disease.

## Materials and methods

### Strains and culturing conditions

The BY4741 (S288C) strain was used throughout this work. A list of yeast strains used in this study can be found in S3 Table. The GFP collection was used for the phenomic screening [54]. Additional GFP-tag and deletion mutants were taken from the GFP collection [54] or deletion mutant array (DMA) (GE, CAT# YSC1053) or were made by PCR-mediated insertion/deletion [99]. All strains listed (S3 Table) were confirmed by PCR. Yeast were cultured at 30°C in YPD, unless otherwise specified. Yeast cultures were grown by shaking in YPD (1% yeast extract, 2% peptone, 2% dextrose) at 30°C unless otherwise stated. In preparation for experiments, yeast were grown overnight in YPD before being diluted to an OD600 of 0.1 (or 0.15 for *eaf1Δ*) in the morning and grown to mid log (OD600 = 0.5–0.8).

### High content screen

A focused mini array of 407 metabolic-associated GFP-tagged genes were extracted from the GFP library (*MATa* ORF-GFP::HIS) [54]. The genes were selected based on literature review and the Saccharomyces Genome Database (SGD) (List of Genes in S1 Table) and fell into categories relating to metabolic processes, gluconeogenesis, changes in regulation dependent on glucose, glucose import, cAMP, and AMPK. Using synthetic genetic array technology [53] and the Singer HTP robotic platform, the GFP fusion metabolic strain mini array was mated to a WT (*ura3Δ*::NAT) or *eaf1Δ* (*eaf1Δ*::NAT) query strains to create both WT and *eaf1*Δ GFP fusion arrays as previously described [50].

Strains generated for the focused high throughput microscopy screen were grown overnight and diluted to 0.1 in the morning. Once they had reached log-phase (OD600 = 0.4–0.7), the cells were collected and diluted in SC media before being plated onto concavalin A-treated 96-well imaging plates. Plated cells were centrifuged and washed twice with SC media. Fluorescent images were taken using a CellVoyager CV1000 disk confocal microscope (Yokogawa Electric Corporation, Musashino Tokyo, Japan). All images were initially captured at a 300 ms exposure for the GFP channel, 30 z-stacked images were taken using the GFP signal with 30 corresponding brightfield images. Two fields of view with at least 35 cells each were captured for each strain (most fields had in the range of 75–200 cells). Images were manually compared for differences in GFP signal between the WT and *eaf1Δ* backgrounds of each GFP-tagged protein by three independent researchers. Each WT- *eaf1Δ* pair was assessed for changes in where the GFP signal was localized and for obvious abundance differences based on GFP intensity in the cell. Additionally, the overall intensity of the WT and *eaf1Δ* images were compared using an in house macro-based batch analysis in ImageJ comparing mean grey value per cell. Two fields of view were analyzed for each strain. Cells were highlighted as region of interest (ROIs) using the brightfield image to define the cell limits and those ROIs were used to measure the GFP signal intensity per cell. The mean grey value per cell was measured as it removed issues with differences in cell size between the WT and *eaf1Δ* strains. The mean grey value of the measured cells was then averaged for each image and the average mean cell intensity of the *eaf1Δ* image was divided by the average mean cell intensity of a paired WT image. This gave a measurement of the change in the intensity of the GFP signal of each protein in an *eaf1Δ* cell relative to WT cell. Changes in intensity were ranked and primary hits were selected as strains having a greater than 1.3-fold increase or 0.7-fold decrease in intensity in the *eaf1Δ* relative to WT. The top 70 ranked images from the manual analysis were also reanalyzed a second time using the same batch analysis comparison of the mean GFP intensity per cell. If changes were detected through the manual scoring or the intensity measurements, they were reconfirmed in another round of microscopy, and top consistent changes were selected resulting in the confirmed set. Localization of the proteins and their associated changes were categorized based on the well characterized WT localization of GFP-tagged proteins in the CYCLoPs database and literature review [50,100].

### Microscopy

All additional microscopy was done using a Leica fluorescent microscope (DMI6000; Leica Microsystems) equipped with a high-performance camera (Hamamatsu), DG4 light source (Sutter Instruments) and Volocity 4.3.2 software (PerkinElmer). Strains were grown to mid log, spun down to collect live cell pellet and resuspended in SC media containing glucose for live GFP and brightfield imaging. As indicated, cells were stained with DAPI (Sigma) prior to spinning down culture. DAPI was added to the liquid culture at a concentration of 1 μg/mL plus 0.1% Triton X-100 (CSH protocols).

Mitochondrial volume was assessed using the MitoMap plugin for ImageJ [65]. Images were corrected to scale dimensions using SetScale. Individual cells were selected and analyzed with the MitoMap plugin [65]. This plugin identifies pixels highlighted with the Cit1-GFP signal and quantifies the volume and surface area of the input mitochondria, for our analysis volume was used. For all quantifications a minimum of 50 cells were analyzed per replicate.

To test mitochondrial response to ethanol, yeast were grown overnight, diluted to OD600 of 0.1 in Yeast Peptone media supplemented with 3% ethanol, a non-fermentable carbon source, and grown to mid log phase before imaging by microscopy. For the fragmentation assay, Hydrogen peroxide was added to mid log day cultures at a final concentration of 4 μM and the cultures were returned to the incubator for 30 minutes before analysis by fluorescent microscopy as described above.

Bcy1-GFP fluorescence was first quantified manually by percent cells with prominent nuclear localization. Additionally, a profile of Bcy1-GFP intensity was measured using ImageJ Software and compressed z-stack images [101]. A 60 unit (6.23 μm) long line was placed across the nucleus of the cell (centred on the nucleus), and Plot Profile analysis was performed to give the fluorescent intensity value of pixels along the line. This was repeated for three biological replicates of 25 cells for each sample. The mean of all replicates was plotted on a representative graph for sample comparison.

### Serial dilution spot assays

Cultures were grown to mid log in YPD before being diluted to an OD600 of 0.1 and three 10-fold serial dilutions (0.01, 0.001, 0.0001). 5 μL of the serial dilutions were spotted onto the indicated plates. To make drug plates, a stock solution of antimycin A (Sigma) was created in 95% Ethanol and was used to create final plates with a concentration of 5 ng/mL. Iodine staining was performed after dot assay growth as described below.

## Glycogen assays

For analysis by iodine staining, spot assays were prepared as above and iodine glycogen assays were performed as previously described [102]. After 24 hours of growth on plain YPD, plates were inverted over iodine crystals for 3–5 minutes, exposing the yeast to the vapours and causing them to stain relative to the abundance of glycogen.

Quantitative measurements of glycogen were assessed by extracting glycogen from cells and treating extracts with amyloglucosidase. This enzyme breaks down glycogen into glucose which can then be measured directly. Cultures of yeast were grown in 100 mL to mid log and a volume of yeast equivalent to 25 ODs was spun down to collect a yeast pellet. Pellets were washed twice in water prior to being flash frozen in liquid nitrogen and stored at -80°C. Pellets were thawed in 500 uL of 0.25 M Na2CO3, vortexed, and boiled at 95oC for 4h. Glycogen (Sigma) was used as a control. The pH of the samples was adjusted by adding 300ul of 1M acetic acid and 1200 ul 0.2M NaOAc pH 5.2. Half of the sample was kept as the no enzyme control and to the other half 20 ul of 20mg/ml amyloglucosidase (Sigma, 10115, 70U/mg protein) was added prior to an overnight incubation at 57°C overnight.

The digested samples were centrifuged, and supernatants were collected. The glucose released by digestion in each sample was diluted and analyzed using the Glucose Colorimetric/Fluorometric assay kit (Biovision, K606) according to the manufacturer’s protocol.

### Growth curve analysis

Cultures were grown to mid log in YPD before being diluted to an OD600 of 0.1 in YPD. 200uL of this dilution was plated into a BioScreen C honeycomb plate. The plate was incubated at 30°C for 48h in a BioScreen C plate reader which collected OD600 readings every 15 minutes. Growth curve data was averaged for 3 technical replicates then 3 biological replicates and plotted for the first 24 hours. The doubling time for each strain was calculated based on the slope of the log (OD600) curve during log growth of the strain (between 3 and 7.5 hours). Doubling time = Δt*Log (2)/ (Log (OD600@t2/OD600@t1)).

### Quantitative western blot

Yeast strains were grown overnight in YPD and diluted into 50 mL cultures in the morning to an OD600 of 0.1. Cells were harvested at mid log and whole cell extracts were collected as previously described [20]. Briefly, cells were collected by centrifugation and pellets were flash frozen in liquid nitrogen and stored at -80°C. The cell pellets were thawed in 3x the pellet volume of lysis buffer (20 mM HEPES, pH 7.4, 0.1% Tween 20, 2 mM MgCl_2_, 300 mM NaCl, protease inhibitor cocktail [P-8215; Sigma]) and lysed using bead beating 6 times for 1 minute with an equal volume of glass beads (Fisher 35–5350). Whole cell extracts were collected by centrifugation and quantified using a Bradford assay. In the case of glucose starvation, cultures were grown to mid log, spun down and washed 2x in YP (glucose free) media and resuspended in YP for 1 hour of growth at 30°C before collecting pellets and freezing as above.

A standard curve for each protein was performed to determine the optimal amount of protein to be loaded on TGX quantitative gels for western blot [103]. For Gsy2-GFP, 40 μg of protein was found to be optimal, so this was loaded onto TGX gels, run, and a standard western blot protocol for TGX gels was followed. Following electrophoresis, the gel was activated and visualized by UV before being transferred onto nitrocellulose (Bio-Rad 1620112). Total protein was imaged using UV. For GFP blots, the blots were blocked in 5% skim milk in PBS-T (phosphate buffered saline + 1% Tween 20) before incubation overnight with 1: 500 dilution of anti-GFP primary antibody (Sigma-Aldrich 11874460000) in 5% milk PBS-T. Next blots were washed 3X in PBS-T, incubated for 1h with goat-anti-mouse secondary antibody (BioRad; cat. # 170–6516) at 1: 2500 in 5% milk PBS-T and washed 3 times. A similar procedure was followed for the PKA assay but blocking and antibodies were done in 3% Bovine Serum Albumin (BSA Sigma-Aldrich A7906). For PKA activity 80 μg of protein was run and the Phospho-PKA Substrate (RRXS*/T*) (Cell Signaling 100G7E) primary was used followed by a goat-anti-rabbit secondary antibody (BioRad; cat. # 170–6515). Finally, blots were incubated with Clarity Western ECL substrate (Bio-Rad 170–5061) and imaged using the Bio-Rad ChemiDoc imaging system. Relative protein abundance of our proteins of interest was quantified using the Image Lab software (Bio-Rad) and each band was normalized to the total protein signal of the lane then calculated relative to the WT signal of the protein.

### qPCR

Yeast were prepared in YPD and 30 mL of OD600 = 0.5 cells were pelleted and washed with water before flash freezing in liquid nitrogen. Cell pellets were thawed in an equal volume of lyticase reaction solution (1.2 M sorbitol + 1 mg/mL lyticase (Sigma)) then incubated at 30°C for 30 min. Cell lysis and RNA purification were then performed using the Ambion PureLink RNA Mini Kit according to manufacturer’s instructions. Briefly, cells were lysed by vortexing in lysis buffer with 2-mercaptoethanol and extracts collected following centrifugation. Ethanol was added to disrupt enzymatic function and RNA was purified and collected using a spin cartridge. RNA extracts were then treated with DNase I for 1 h at 37°C before performing a phenol/chloroform/isoamyl extraction and measuring RNA concentration by nanodrop. At this point the quality of the RNA was assessed by agarose gel. 2 μg of the mRNA was reverse transcribed using the High Capacity cDNA Reverse Transcription Kit (Applied Biosystems). Serial dilutions of the cDNA product were performed and a pooled standard curve to determine optimal dilution for each transcript type. mRNA transcript levels were measured using the SsoFast EvaGreen Supermix for qPCR (Bio-Rad) with the appropriate primer pairs (S4 Table). The data for each experiment was normalized to the chosen internal control (*TDH3* mRNA) and then compared to WT using the ΔΔCt method.

### Seahorse assays

Seahorse assays were performed as previously described [104]. Briefly, Yeast cultures were grown overnight in YPD and in the morning they were split and diluted to an OD600 of 0.1 in YPD or 0.25 in YPE (Yeast Peptone Ethanol 3%), grown to mid log (OD600 of 0.5–0.7) prior to cells being pelleted (3000 RPM for 3 min) and resuspended into yeast seahorse assay media (0.167% yeast nitrogen base, 0.5% ammonium sulfate, and 3% ethanol or 2% dextrose respectively). Cells were counted and diluted to a concentration of 2.8 x 10^6 cells/mL in yeast seahorse media. 180 μL of the cell dilutions were used to seed the wells of a Seahorse XF96 cell culture microplate (Agilent) that had been pre-treated with poly-l-lysine to enable cell adhesion (MilliporeSigma). A minimum of 8 technical replicates were performed for each experiment. The seeded plate was centrifuged at 500 RPM for 3 min to promote yeast adhesion and the plate was rested for 30 min at 30°C. A soaked and calibrated Seahorse XF96 Sensor Cartridge was prepared with 0.5% sodium azide (Sigma) injection in chamber A before loading into the Seahorse XF96 analyzer (Agilent) and measurement of oxygen consumption rate (OCR) of the yeast in the cell culture microplate. Measurements were performed at 30°C. Three basal OCR measurements were taken for 3 minutes each with 1.5 minutes of mixing between measurements. Sodium azide was then programmed to be injected and three additional OCR measurements were performed for 3 min with 1.5 minutes of mixing between.

### Acetylation mimics/CRISPR-Cas9

Point mutations on Bcy1 at the lysine 313 (K313) site were performed by targeting with CRISPR-Cas9 to cut the DNA and introducing a homologous strand of DNA with the mutation of interest. The lysine 313 site of Bcy1 was mutated to a Glutamine (Q) or Arginine (R) to represent an unacetylated and an acetylated form of Bcy1 (K313Q and K313R).

The general DiCarlo *et*. *al*. protocol was followed with minor adjustments. Briefly, the Cas9 plasmid, p414-TEF1p-Cas9-CYC1t [105], was transformed using lithium acetate into the Bcy1-GFP, Bcy1-GFP *eaf1Δ*, Cit1-GFP, and cit1-GFP *eaf1Δ* strains and maintained by growth on SC-LEU. In parallel to account for problems with off target effects, two different PAM sequences targeting the Bcy1-K313 area were cloned into the guide RNA plasmid, p426-SNR52p-NotI (gRNA)-SUP4t by PCR amplification. These sequences were selected from DiCarlo and colleague’s list [105]. Following PCR, the resulting plasmid was treated with DPN1, transformed into DH5α *E*. *coli* for amplification, and sent for sequencing at the TCAG facility (SickKids) to confirm guide RNA incorporation. Double stranded DNA homologous to the area to be cleaved but containing the mutations to the K313 and the NGG PAM site was prepared by ordering single stranded oligos and annealing them at 100°C and slowly ramping down the temperature. The dsDNA for homologous repair and the corresponding guide RNA plasmid were transformed using lithium acetate into the strains pre-prepared with the Cas9 expression plasmid. Transformed cells were selected on SC-LEU-URA and positive colonies were collected, genomic DNA extracted, and a PCR of the area of interest was performed, which was then sent for sequencing at the TCAG Facility (SickKids, Toronto) to confirm the mutation in the genome.

### Statistical analyses information

Statistical analyses were performed using GraphPad Prism. When comparing two groups a student’s t-test was performed. In the case of more than two groups a 2-way ANOVA was performed, and individual pairs were compared by a Tukey’s multiple comparison test comparing each mean to the mean of every other group. For all analyses a p < 0.05 to define significance was used.

## Supporting information

S1 Fig Summary of screen protocol. (A) We selected 407 metabolic genes from the yeast GFP collection where each ORF is tagged with GFP and the *HIS3MX* to create a metabolic protein mini array. This mini array was crossed to WT and *eaf1*Δ *MAT* alpha query strains and an SGA protocol was followed to create two GFP mini arrays. The protein localization of each metabolic protein was compared between the WT and *eaf1Δ* arrays by high throughput microscopy. (B) A flow chart tracking the strains and markers that were used and produced during our SGA protocol and the selection medias that were used along the way.
(TIFF)

S2 Fig Changes in metabolic protein GFP intensity between and *eaf1Δ* and WT. The mean intensity of GFP signal per cell of each strain was compared between the WT and the *eaf1Δ*. For each GFP-tagged metabolic protein, two field of view images were taken for each the WT and *eaf1Δ* strain using both brightfield and the GFP channel. The *eaf1Δ* average intensity per cell was divided by the paired WT average intensity per cell to give a relative change in GFP signal (*eaf1Δ* cell intensity/WT cell intensity). The relative change was then ranked and plotted using MatPlotLib. Proteins which had a larger than 1.3 or less than 0.7-fold change in were deemed primary hits (outside of the horizontal red lines). All raw intensity measurements and summarized changes with gene names are available in S2. Blue spots are quantifications of the first pass of the screen and orange X points are quantifications of the secondary assessment of primary hits.
(TIFF)

S3 Fig Example images of the 23 proteins that were confirmed to change between WT and *eaf1Δ* in our GFP Screen. Scale bar = 10 μm.
(TIFF)

S4 Fig NuA4 plays a role in the regulation of glycogen synthesis. (A) Representative images of WT and *eaf1Δ* cells expressing Gdb1-GFP, Gsy1-GFP and Gsy2-GFP taken from our screen. (Scale bar = 10 μm). (B) Representative western blot of WT or *eaf1Δ* whole cell extracts containing Gsy1-GFP, Gsy2-GFP, and Gdb1-GFP. These show that the abundance of each of these proteins increases in an *eaf1Δ* relative to WT. (C) The glycogen content of WT, *eaf1Δ*, and the temperature sensitive *esa1-ts* mutant were assessed at 3 temperatures using an iodine staining procedure, darker colour is indicative of increased glycogen content. Yeast were spotted onto YPD in 10-fold serial dilutions and grown for 24 h at the designated temperature prior to exposure to iodine crystals. Image is representative of three biological replicates.
(TIFF)

S5 Fig *eaf1Δ* strains have a growth defect which is reversed upon deletion of *BCY1*. (A) Growth curves were produced by measuring OD600 over 24 hours for each of the strains using a BioScreen C plate reader. Three biological replicates were performed of which one representative set is shown here. (B) Doubling time, the number of hours it takes for the yeast culture to double, was calculated using average slope of the log OD growth curve between 3 and 7.5 hours for three biological replicates. ANOVA analysis was performed with a Tukey’s multiple comparison test comparing pairs of means. * = p < 0.05, n.s. = non-significant, relevant significance bars shown.
(TIFF)

S6 Fig Mitochondrial structure of WT and *eaf1*Δ assessed using Aco2-GFP and MitoLoc. Yeast with integrated Aco2-GFP and yeast that contained the MitoLoc Plasmid [65] were prepared to mid-log in YPD for microscopy. Mitochondrial structure was assessed in WT and *eaf1*Δ backgrounds with Aco2-GFP or MitoLoc. Scale bar = 10 μm.
(TIFF)

S7 Fig Deletion of *EAF1* affect the size of the cell, measurements of cell size. Cell volume was approximated by taking 2 measurements of cell diameter using ImageJ on scale images, averaging them, and using half that diameter in the 4/3πr^3^ formula. This approximates cell volume based on a sphere of the yeast’s average diameter. (A) Measurements of cell volume for WT and *eaf1Δ* at 30°C, corresponds with Fig 3. (B) Measurements of cell volume for WT and targeted double mutants, corresponds with Fig 5. (C) Measurements of cell volume for WT and *bcy1* CRISPR-Cas9 mutants, corresponds with Fig 7.
(TIFF)

S8 Fig Raw mitochondrial volume measurements. The raw mitochondrial volume of the mitochondria was quantified based on the Cit1-GFP fluorescence and using the MitoMap plugin for ImageJ for 3 biological replicates and at least 50 cells per replicate were analyzed [64]. (A) Raw measurements of mitochondrial volume per cell (μm^3^) for WT and *eaf1Δ* at 30°C, corresponds with Fig 3. (B) Raw measurements of mitochondrial volume per cell (μm ^3^) for WT and targeted double mutants, corresponds with Fig 5. (C) Raw measurements of mitochondrial volume per cell (μm ^3^) for WT and *bcy1* CRISPR mutants, corresponds with Fig 7.
(TIFF)

S9 Fig Mitochondrial morphology of the temperature sensitive *ESA1* mutant. (A) The mitochondrial morphology of WT and the *esa1-ts* mutant was assessed after being grown to early log at 25°C and temperature shifted to 33°C for 2h with the mitochondrial marker Cit1-GFP. The mitochondrial fraction was quantified based on the Cit1-GFP fluorescence using the MitoMap plugin for ImageJ which was then divided by the average total cellular volume of the strain for 3 independent biological replicates. Images are representative of 3 independent biological replicates and at least 50 cells per replicate were analyzed per replicate for quantification. Scale bar = 10 μm. An unpaired T-test was used to compare groups. (B) WT and *esa1-ts* yeast were grown overnight and in day cultures at 25°C before being transitioned to the restrictive temperature of 33°C for 2h. Mitochondrial structure was assessed using Aco2-GFP. (C) Cell volume measurements of temperature shifted WT and *esa1-ts*. Cell volume was approximated by taking 2 measurements of cell diameter using ImageJ on scale images, averaging them, and using half that diameter in the 4/3πr^3^ formula. This approximates cell volume based on a sphere of the yeast’s average diameter. (D) Raw measurements of mitochondrial volume per cell (μm ^3^) for WT and *esa1-ts*.
(TIFF)

S10 Fig Deletion of *HAP4*, a transcription factor involved in mitochondrial biogenesis, does not reverse the elongation of the mitochondria seen in an *eaf1Δ*. Cit1-GFP was used as a marker of the mitochondrial structure in *hap4*Δ and *eaf1Δ hap4Δ* mutants.
(TIFF)

S11 Fig TPK protein localization does not change between the WT and *eaf1Δ*. (TIFF)

S12 Fig Three replicate western blots showing PKA activity is altered in an *eaf1Δ*. Representative and quantification shown in Fig 9. PKA substrate phosphorylation under (A) log growth in glucose rich conditions and (B) Glucose starved conditions was assessed by quantitative western blot analysis using whole cell extracts from WT and *eaf1Δ* strains.
(TIFF)

S1 Table List of screened strains, top 70 changes, and final 23 changes. (XLSX)

S2 Table Classification of the 23 proteins that were confirmed to change between WT and *eaf1*Δ in our GFP screen. (DOCX)

S3 Table List of yeast strains used in this work. (XLSX)

S4 Table List of primers used in this work. (XLSX)
