# Supplementary figures and images for: Phenomic screen identifies a role for the yeast lysine acetyltransferase NuA4 in the control of Bcy1 subcellular localization, glycogen biosynthesis, and mitochondrial morphology
Source: PLoS Genet. 2020 Nov 30;16(11):e1009220. doi: 10.1371/journal.pgen.1009220 (PMC7728387; doi:10.1371/journal.pgen.1009220)

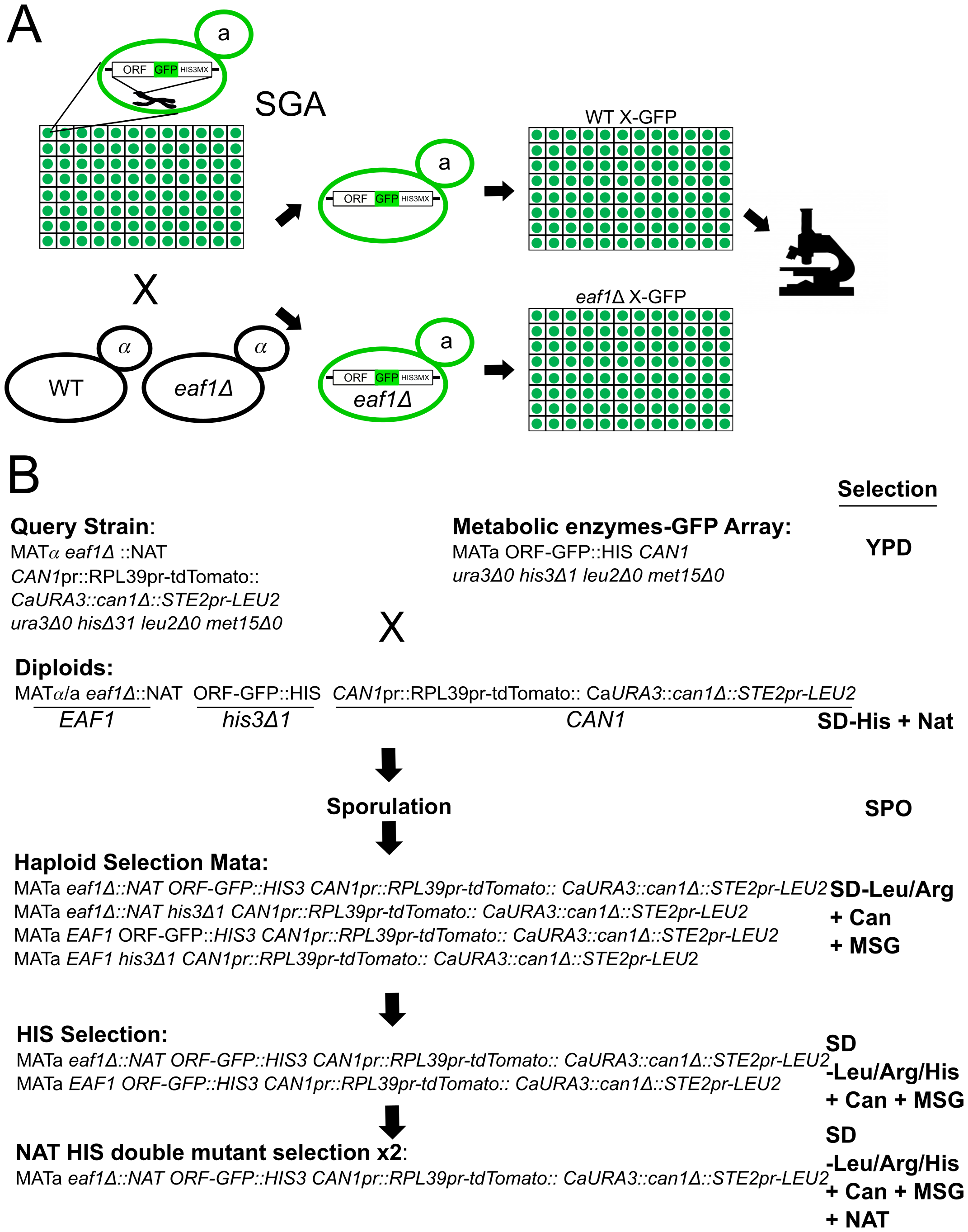

Supplement: S1 Fig — (A) We selected 407 metabolic genes from the yeast GFP collection where each ORF is tagged with GFP and the HIS3MX to create a metabolic protein mini array. This mini array was crossed to WT and eaf1Δ MAT alpha query strains and an SGA protocol was followed to create two GFP mini arrays. The protein localization of each metabolic protein was compared between the WT and eaf1Δ arrays by high throughput microscopy. (B) A flow chart tracking the strains and markers that were used and produced during our SGA protocol and the selection medias that were used along the way. (TIFF) [file pgen.1009220.s001.tiff]

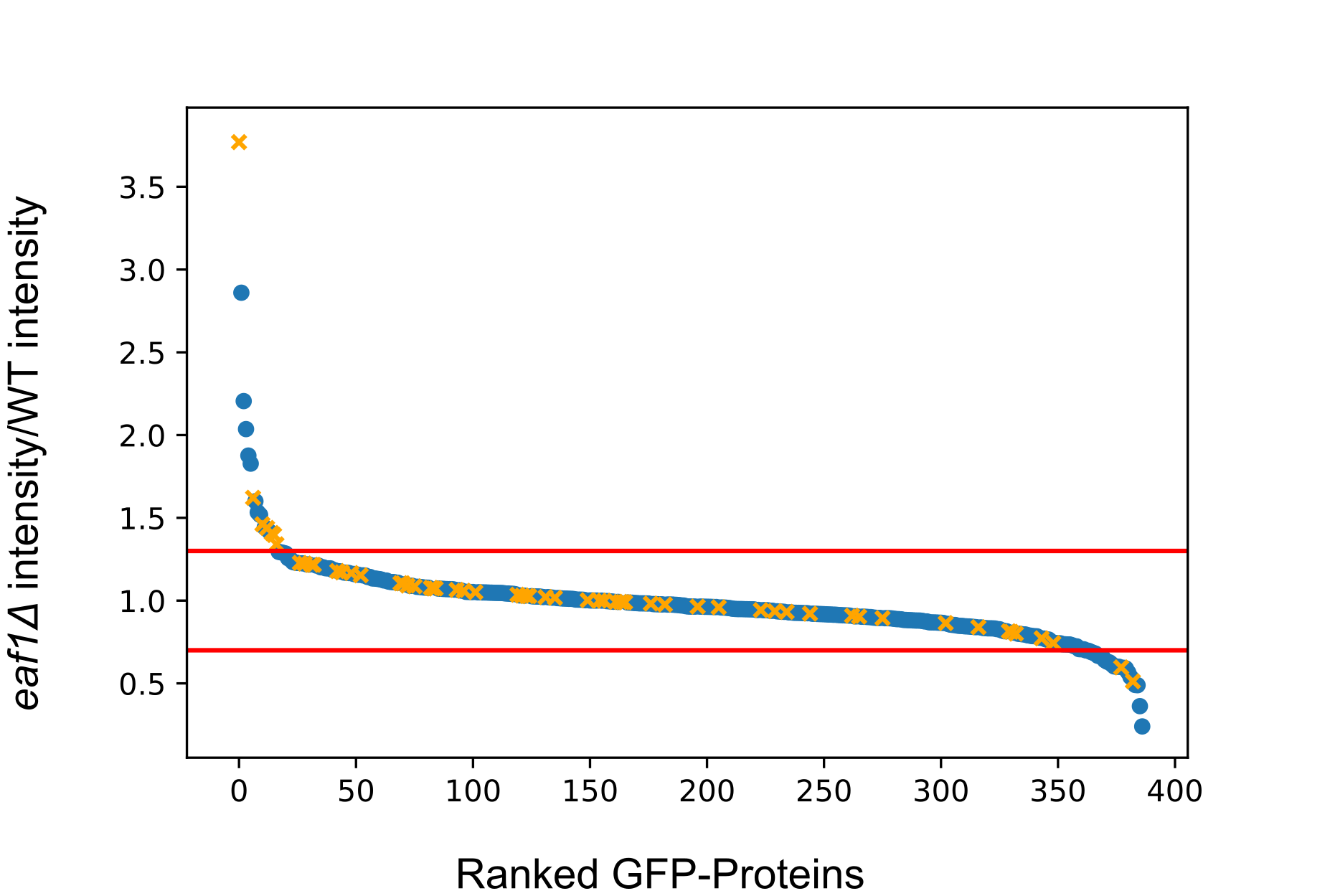

Supplement: S2 Fig — The mean intensity of GFP signal per cell of each strain was compared between the WT and the eaf1Δ. For each GFP-tagged metabolic protein, two field of view images were taken for each the WT and eaf1Δ strain using both brightfield and the GFP channel. The eaf1Δ average intensity per cell was divided by the paired WT average intensity per cell to give a relative change in GFP signal (eaf1Δ cell intensity/WT cell intensity). The relative change was then ranked and plotted using MatPlotLib. Proteins which had a larger than 1.3 or less than 0.7-fold change in were deemed primary hits (outside of the horizontal red lines). All raw intensity measurements and summarized changes with gene names are available in S2. Blue spots are quantifications of the first pass of the screen and orange X points are quantifications of the secondary assessment of primary hits. (TIFF) [file pgen.1009220.s002.tiff]

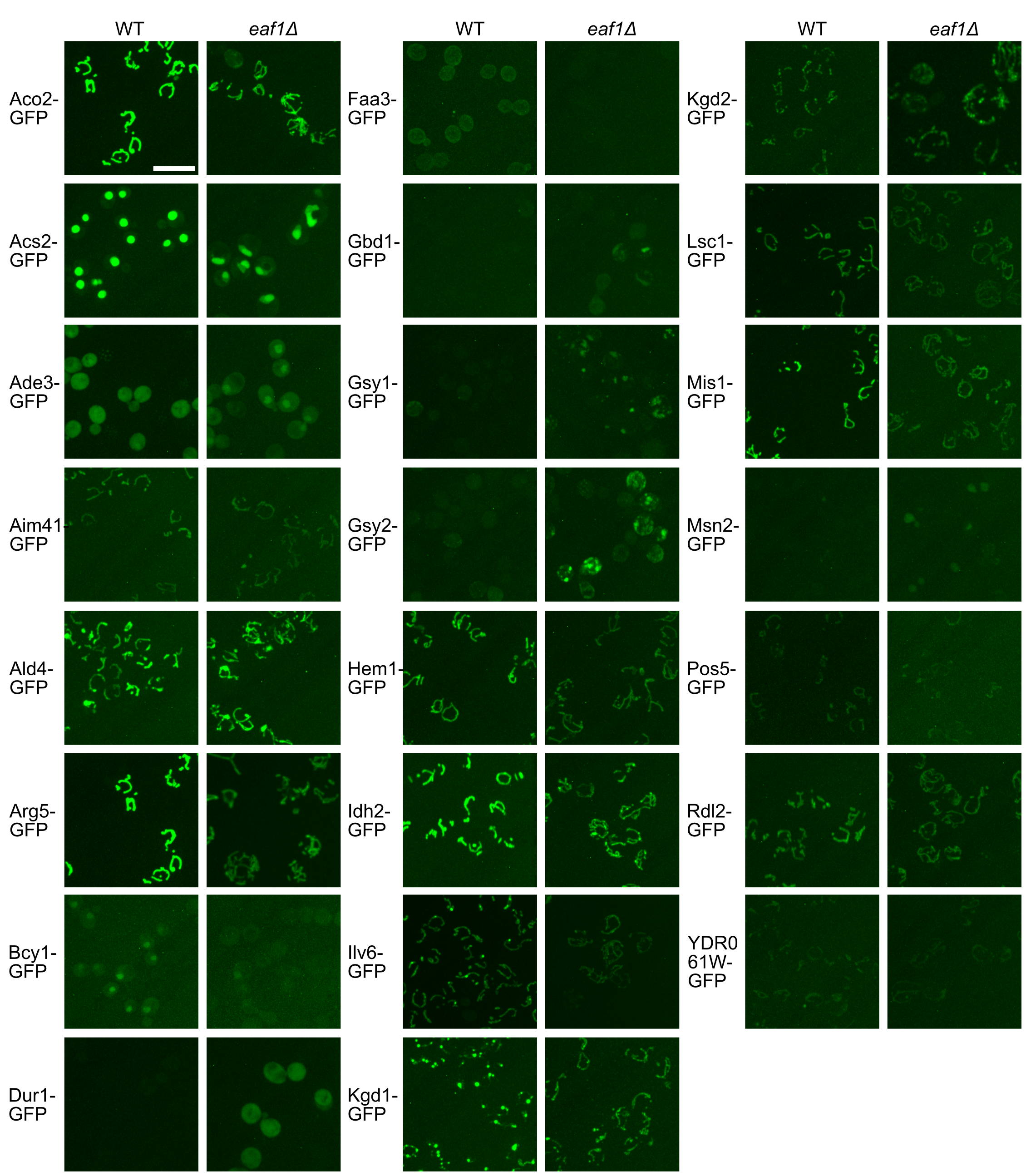

Supplement: S3 Fig — Scale bar = 10 μm. (TIFF) [file pgen.1009220.s003.tiff]

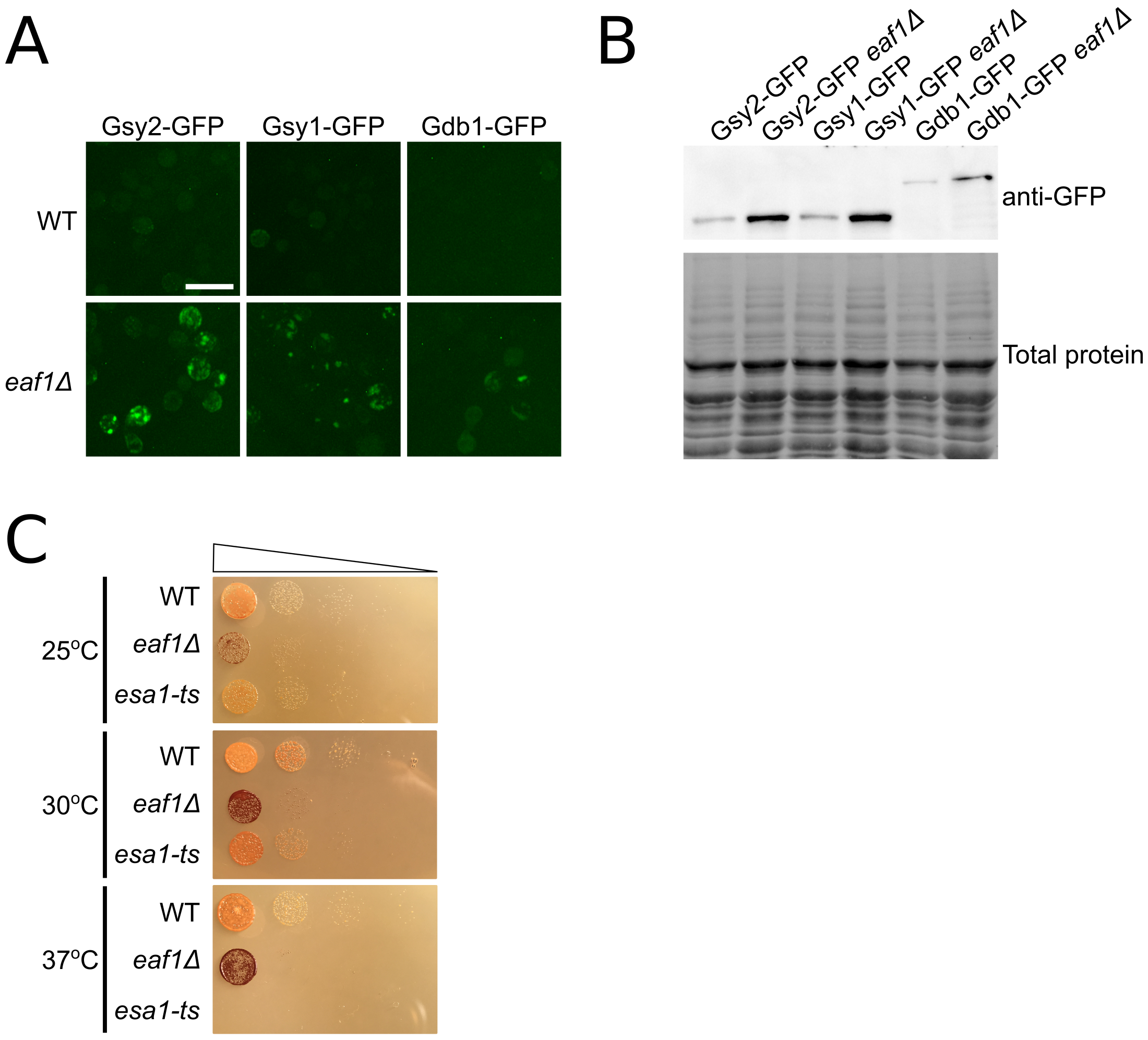

Supplement: S4 Fig — (A) Representative images of WT and eaf1Δ cells expressing Gdb1-GFP, Gsy1-GFP and Gsy2-GFP taken from our screen. (Scale bar = 10 μm). (B) Representative western blot of WT or eaf1Δ whole cell extracts containing Gsy1-GFP, Gsy2-GFP, and Gdb1-GFP. These show that the abundance of each of these proteins increases in an eaf1Δ relative to WT. (C) The glycogen content of WT, eaf1Δ, and the temperature sensitive esa1-ts mutant were assessed at 3 temperatures using an iodine staining procedure, darker colour is indicative of increased glycogen content. Yeast were spotted onto YPD in 10-fold serial dilutions and grown for 24 h at the designated temperature prior to exposure to iodine crystals. Image is representative of three biological replicates. (TIFF) [file pgen.1009220.s004.tiff]

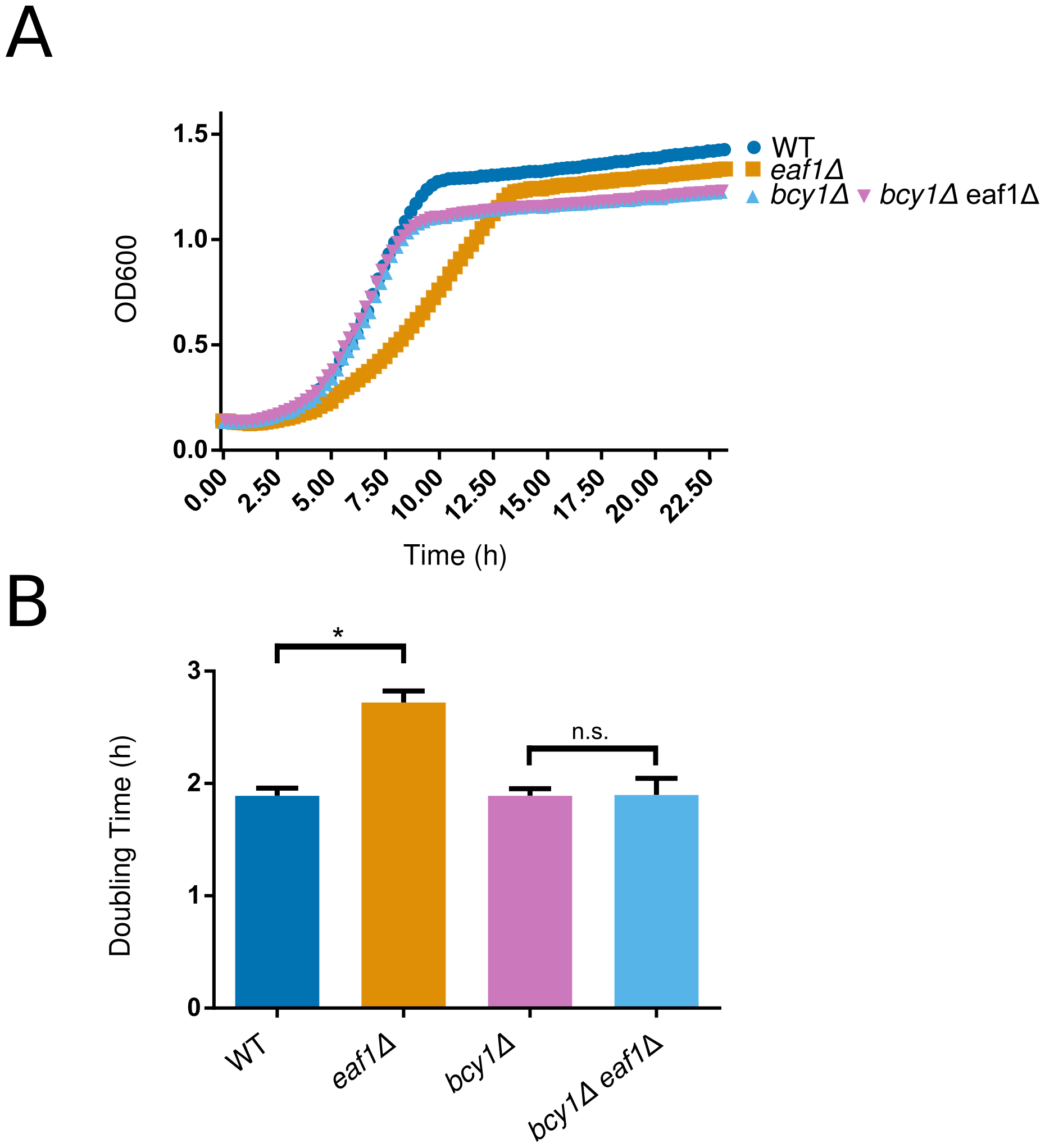

Supplement: S5 Fig — (A) Growth curves were produced by measuring OD600 over 24 hours for each of the strains using a BioScreen C plate reader. Three biological replicates were performed of which one representative set is shown here. (B) Doubling time, the number of hours it takes for the yeast culture to double, was calculated using average slope of the log OD growth curve between 3 and 7.5 hours for three biological replicates. ANOVA analysis was performed with a Tukey’s multiple comparison test comparing pairs of means. * = p < 0.05, n.s. = non-significant, relevant significance bars shown. (TIFF) [file pgen.1009220.s005.tiff]

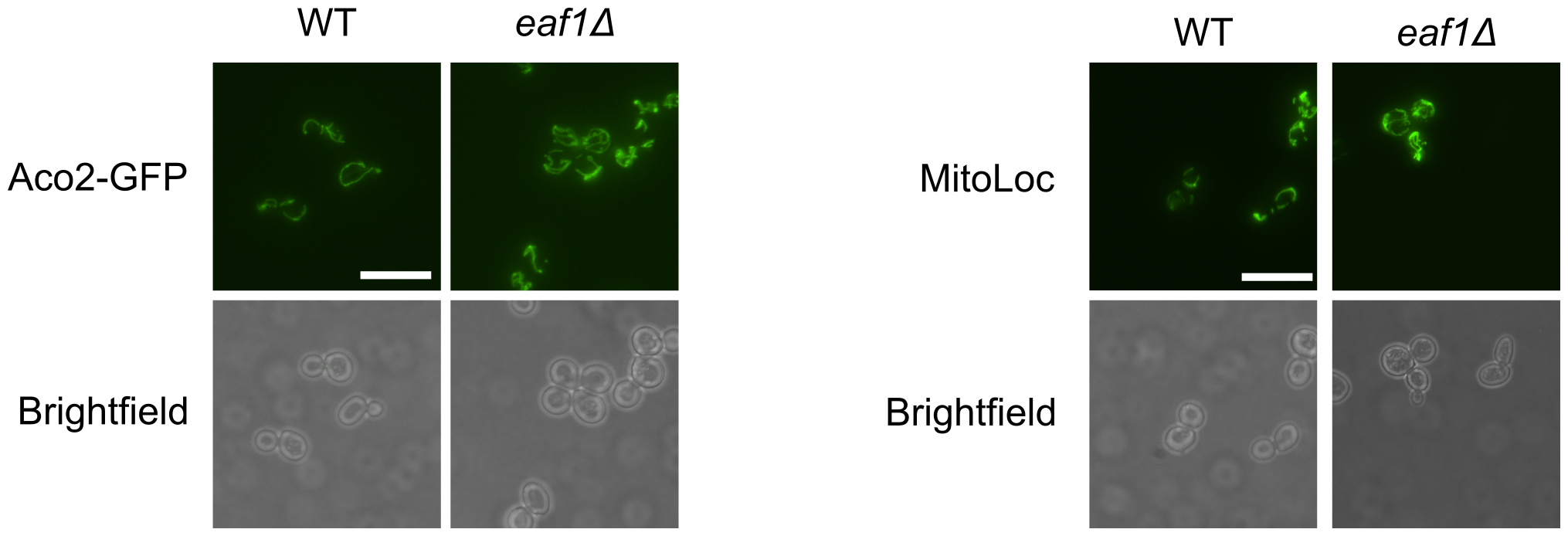

Supplement: S6 Fig — Yeast with integrated Aco2-GFP and yeast that contained the MitoLoc Plasmid [65] were prepared to mid-log in YPD for microscopy. Mitochondrial structure was assessed in WT and eaf1Δ backgrounds with Aco2-GFP or MitoLoc. Scale bar = 10 μm. (TIFF) [file pgen.1009220.s006.tiff]

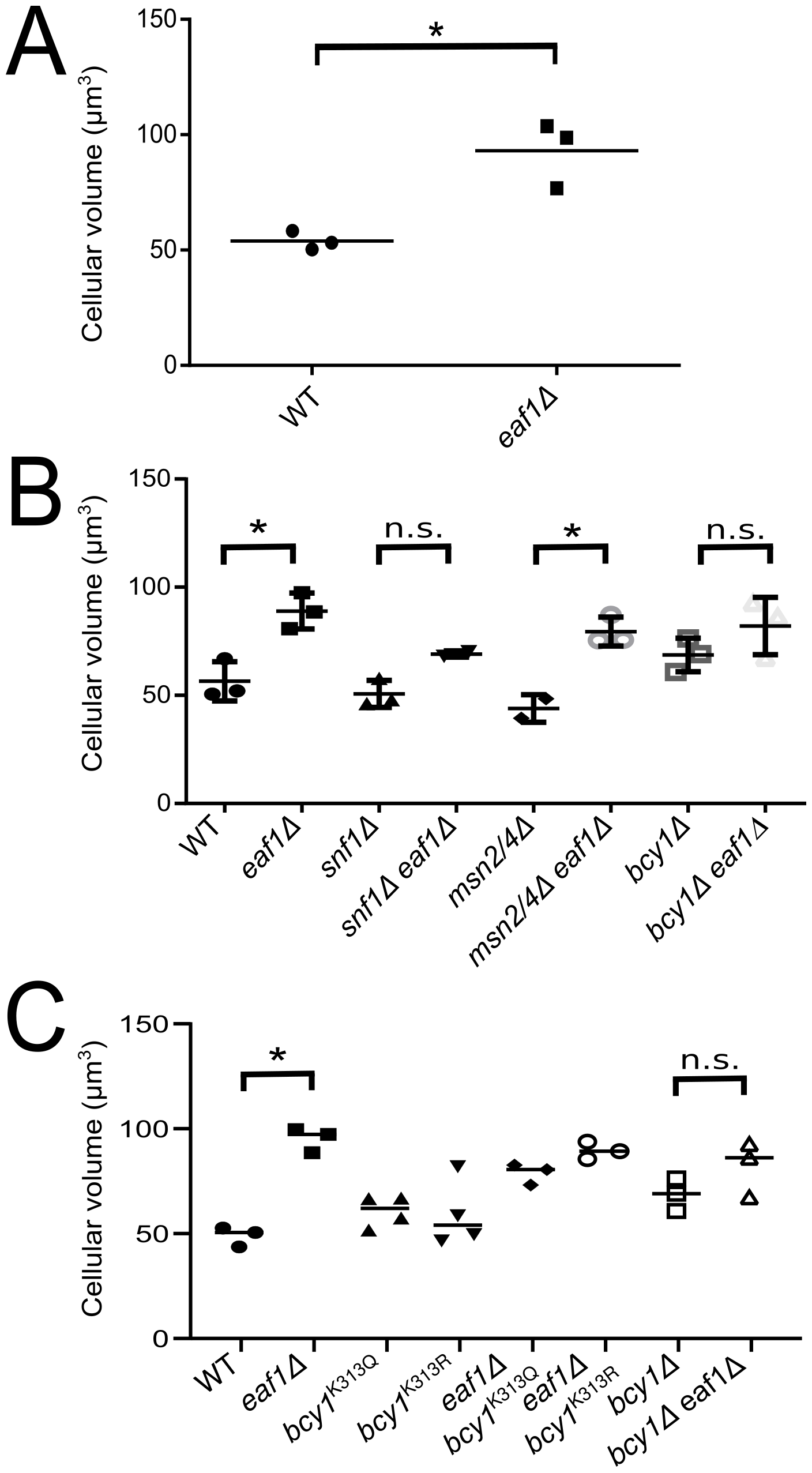

Supplement: S7 Fig — Cell volume was approximated by taking 2 measurements of cell diameter using ImageJ on scale images, averaging them, and using half that diameter in the 4/3πr3 formula. This approximates cell volume based on a sphere of the yeast’s average diameter. (A) Measurements of cell volume for WT and eaf1Δ at 30°C, corresponds with Fig 3. (B) Measurements of cell volume for WT and targeted double mutants, corresponds with Fig 5. (C) Measurements of cell volume for WT and bcy1 CRISPR-Cas9 mutants, corresponds with Fig 7. (TIFF) [file pgen.1009220.s007.tiff]

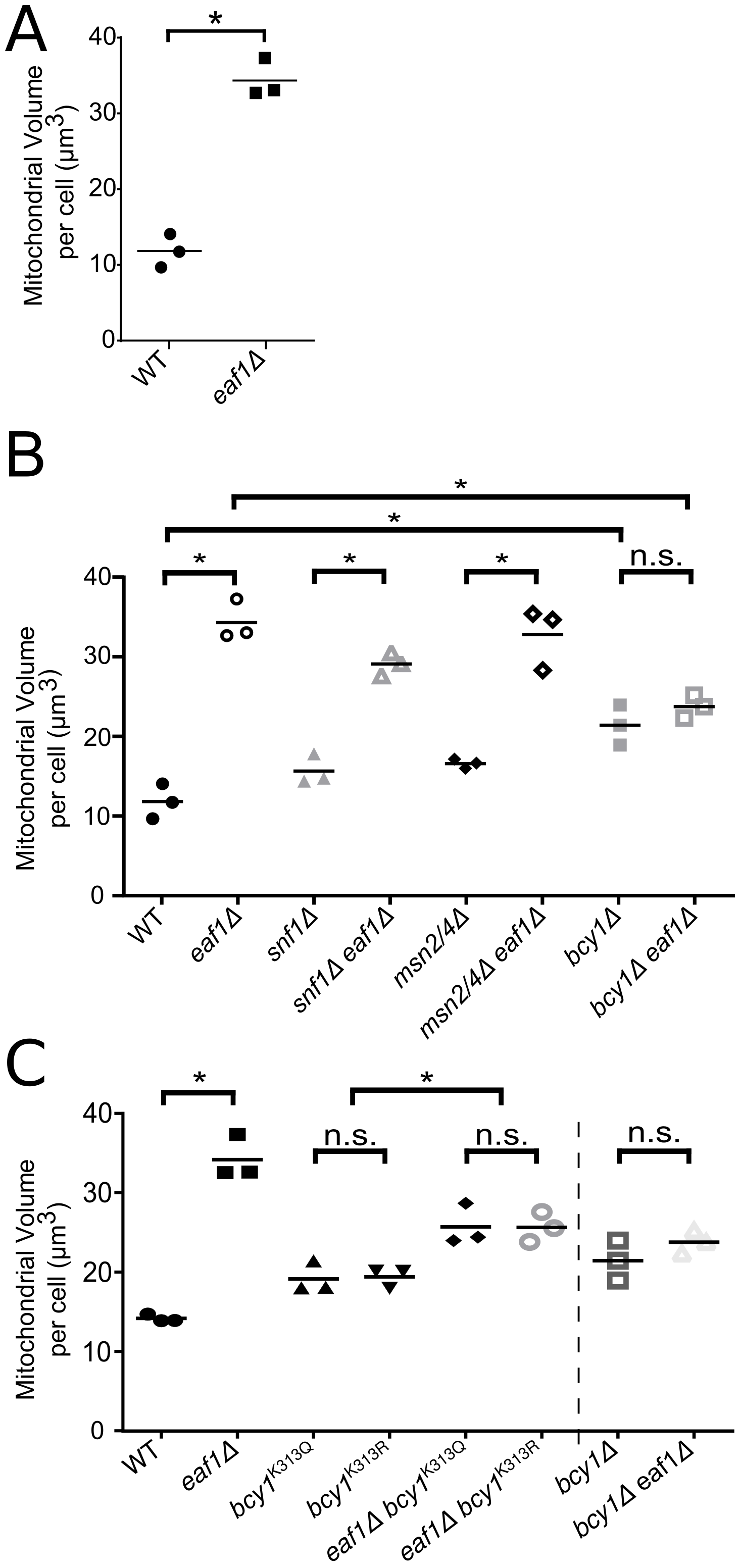

Supplement: S8 Fig — The raw mitochondrial volume of the mitochondria was quantified based on the Cit1-GFP fluorescence and using the MitoMap plugin for ImageJ for 3 biological replicates and at least 50 cells per replicate were analyzed [64]. (A) Raw measurements of mitochondrial volume per cell (μm3) for WT and eaf1Δ at 30°C, corresponds with Fig 3. (B) Raw measurements of mitochondrial volume per cell (μm 3) for WT and targeted double mutants, corresponds with Fig 5. (C) Raw measurements of mitochondrial volume per cell (μm 3) for WT and bcy1 CRISPR mutants, corresponds with Fig 7. (TIFF) [file pgen.1009220.s008.tiff]

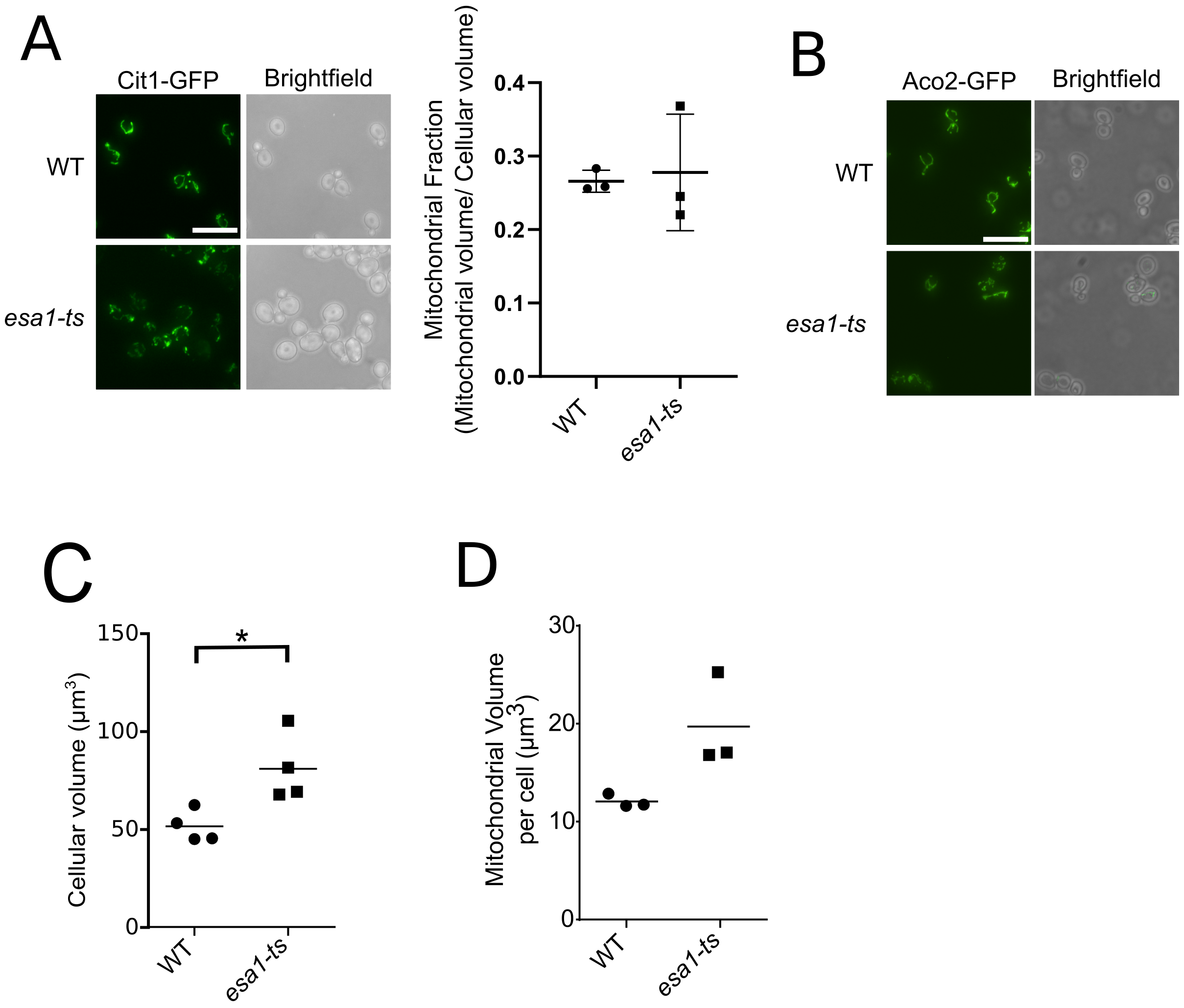

Supplement: S9 Fig — (A) The mitochondrial morphology of WT and the esa1-ts mutant was assessed after being grown to early log at 25°C and temperature shifted to 33°C for 2h with the mitochondrial marker Cit1-GFP. The mitochondrial fraction was quantified based on the Cit1-GFP fluorescence using the MitoMap plugin for ImageJ which was then divided by the average total cellular volume of the strain for 3 independent biological replicates. Images are representative of 3 independent biological replicates and at least 50 cells per replicate were analyzed per replicate for quantification. Scale bar = 10 μm. An unpaired T-test was used to compare groups. (B) WT and esa1-ts yeast were grown overnight and in day cultures at 25°C before being transitioned to the restrictive temperature of 33°C for 2h. Mitochondrial structure was assessed using Aco2-GFP. (C) Cell volume measurements of temperature shifted WT and esa1-ts. Cell volume was approximated by taking 2 measurements of cell diameter using ImageJ on scale images, averaging them, and using half that diameter in the 4/3πr3 formula. This approximates cell volume based on a sphere of the yeast’s average diameter. (D) Raw measurements of mitochondrial volume per cell (μm 3) for WT and esa1-ts. (TIFF) [file pgen.1009220.s009.tiff]

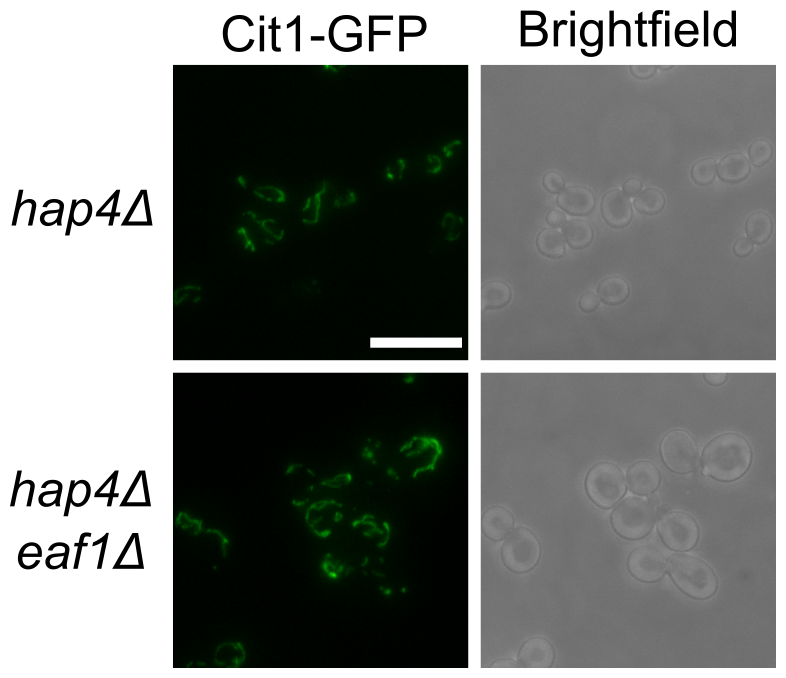

Supplement: S10 Fig — Cit1-GFP was used as a marker of the mitochondrial structure in hap4Δ and eaf1Δ hap4Δ mutants. (TIFF) [file pgen.1009220.s010.tiff]

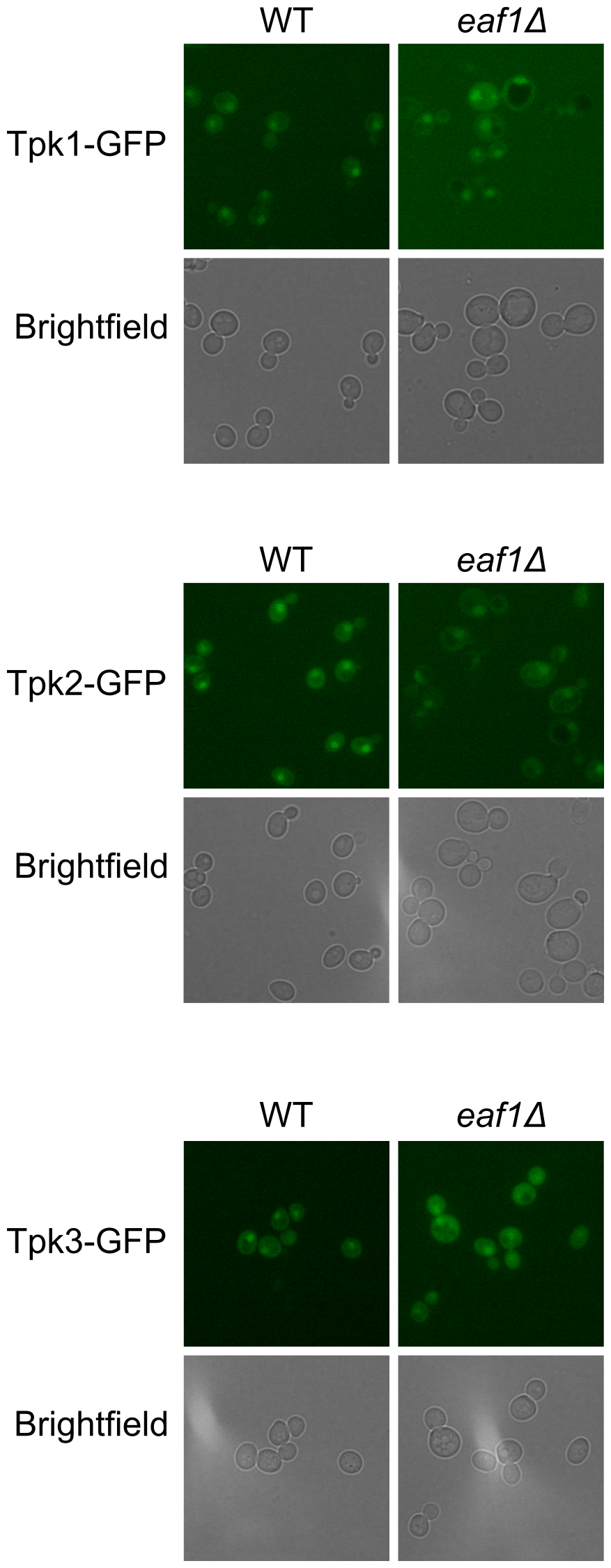

Supplement: S11 Fig — (TIFF) [file pgen.1009220.s011.tiff]

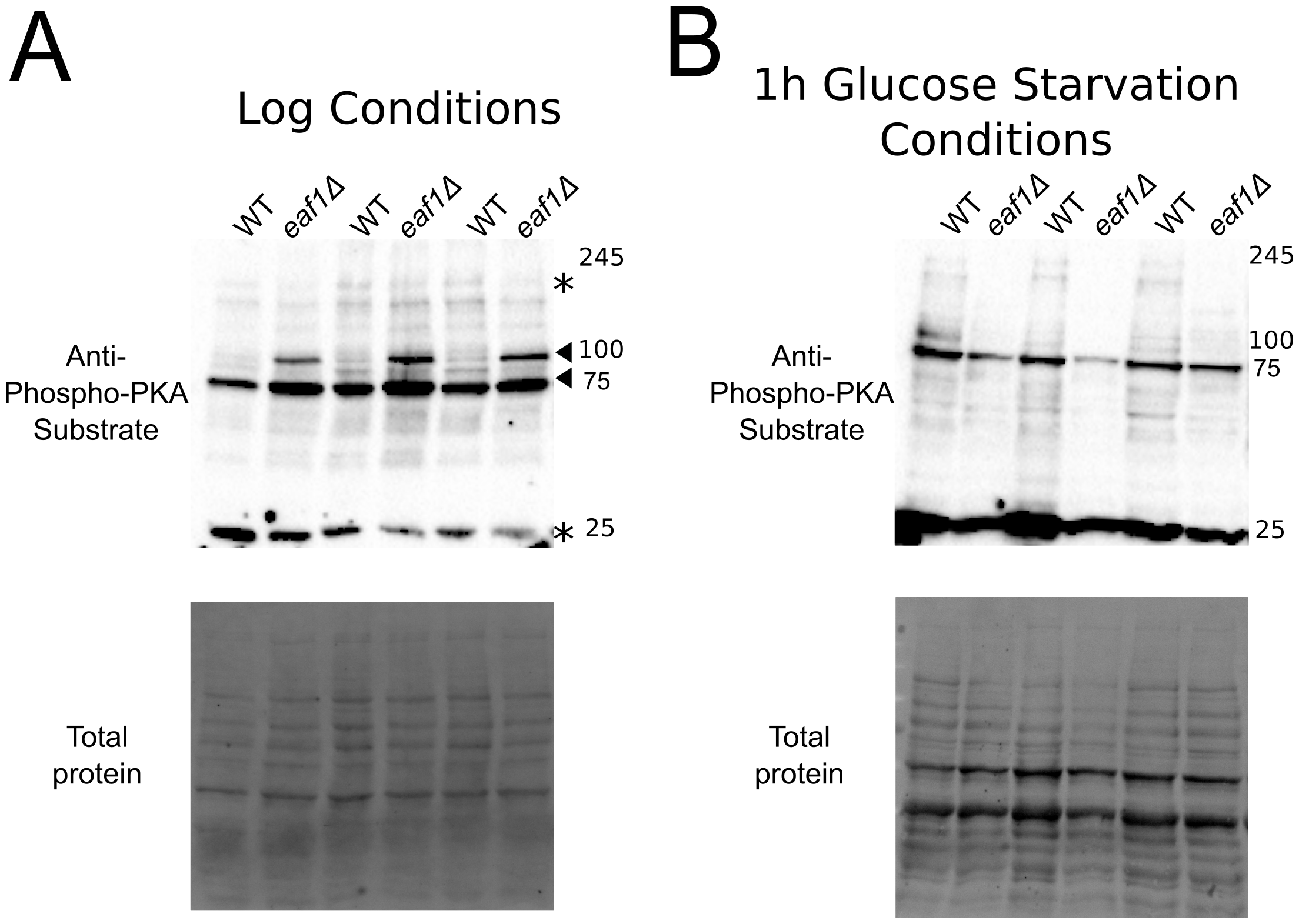

Supplement: S12 Fig — Representative and quantification shown in Fig 9. PKA substrate phosphorylation under (A) log growth in glucose rich conditions and (B) Glucose starved conditions was assessed by quantitative western blot analysis using whole cell extracts from WT and eaf1Δ strains. (TIFF) [file pgen.1009220.s012.tiff]
