# Supplementary material for: Phenomic screen identifies a role for the yeast lysine acetyltransferase NuA4 in the control of Bcy1 subcellular localization, glycogen biosynthesis, and mitochondrial morphology
Source: PLoS Genet. 2020 Nov 30;16(11):e1009220. doi: 10.1371/journal.pgen.1009220 (PMC7728387; doi:10.1371/journal.pgen.1009220)
Supplement: S2 Table — (DOCX) [file pgen.1009220.s014.docx]

| **Changes in protein/GFP signal classification** | **Gene** |
| --- | --- |
| Mitochondrial elongation | *ACO2* |
|  | *AIM41* |
|  | *ALD4* |
|  | *ARG5* |
|  | *HEM1* |
|  | *IDH2* |
|  | *ILV6* |
|  | *KGD2* |
|  | *LSC1* |
|  | *MIS1* |
|  | *POS5* |
|  | *RDL2* |
|  | *YDR061W* |
|  | *KGD11* |
| Cytosol to punctate | *GBD1* |
|  | *GSY1* |
|  | *GSY2* |
| Nucleus to cytosol | *ACS2* |
|  | *BCY1* |
| Cytosol to nucleus | *ADE3* |
|  | *MSN2* |
| Cell periphery to cytoplasm | *FAA3* |
| Increased abundance | *DUR1* |

**S2 Table:** A classification of the 23 genes which were identified with high confidence as changing in protein/GFP signal between WT and *eaf1Δ* yeast. Six categories of changes were identified.
